# Clinical and genetic characterization of a progressive *RBL2*-associated neurodevelopmental disorder

**DOI:** 10.1093/brain/awae363

**Published:** 2024-12-18

**Authors:** Gabriel N Aughey, Elisa Cali, Reza Maroofian, Maha S Zaki, Alistair T Pagnamenta, Zafar Ali, Uzma Abdulllah, Fatima Rahman, Lara Menzies, Anum Shafique, Mohnish Suri, Emmanuel Roze, Mohammed Aguennouz, Zouiri Ghizlane, Saadia Maryam Saadi, Ambrin Fatima, Huma Arshad Cheema, Muhammad Nadeem Anjum, Godelieve Morel, Stephanie Robin, Robert McFarland, Umut Altunoglu, Verena Kraus, Moneef Shoukier, David Murphy, Kristina Flemming, Hilde Yttervik, Hajar Rhouda, Gaetan Lesca, Nicolas Chatron, Massimiliano Rossi, Bibi Nazia Murtaza, Mujaddad Ur Rehman, Jenny Lord, Edoardo Giacopuzzi, Azam Hayat, Muhammad Siraj, Reza Shervin Badv, Go Hun Seo, Christian Beetz, Hülya Kayserili, Yamna Krioulie, Wendy K Chung, Sadaf Naz, Shazia Maqbool, Kate E Chandler, Christopher J Kershaw, Thomas Wright, Siddharth Banka, Joseph G Gleeson, Jenny C Taylor, Stephanie Efthymiou, Shahid Mahmood Baig, Mariasavina Severino, James E C Jepson, Henry Houlden

**Affiliations:** Department of Clinical and Experimental Epilepsy, UCL Queen Square Institute of Neurology, London WC1N 3BG, UK; Department of Neuromuscular diseases, UCL Queen Square Institute of Neurology, London WC1N 3BG, UK; Department of Neuromuscular diseases, UCL Queen Square Institute of Neurology, London WC1N 3BG, UK; Department of Clinical Genetics, Human Genetics and Genome Research Institute, National Research Centre, Dokki, Cairo 12622, Egypt; NIHR Oxford Biomedical Research Centre, Centre for Human Genetics, University of Oxford, Oxford OX3 7BN, UK; Centre for Biotechnology and Microbiology, University of Swat, Charbagh, Swat, Khyber Pakhtunkhwa 19120, Pakistan; University Institute of Biochemistry and Biotechnology (UIBB), PMAS-Arid Agriculture University Rawalpindi, Rawalpindi 46300, Pakistan; Department of Developmental-Behavioral Pediatrics, The Children’s Hospital, University of Child Health Sciences (UCHS-CH), Lahore 54600, Pakistan; Department of Clinical Genetics, Great Ormond Street Hospital for Children NHS Foundation Trust, London WC1N 3JH, UK; School of Biological Sciences, University of the Punjab, Lahore 54590, Pakistan; UK National Paediatric Ataxia Telangiectasia Clinic, Nottingham University Hospitals NHS Trust, Nottingham NG5 1PB, UK; Nottingham Clinical Genetics Service, Nottingham University Hospitals NHS Trust, Nottingham NG5 1PB, UK; INSERM, CNRS, Sorbonne University, Paris Brain Institute, Salpêtrière Hospital/AP-HP, Paris 75013, France; Department of Clinical and Experimental Medicine, University of Messina, Messina 98122, Italy; Unit of Neuropediatrics and Neurometabolism, Pediatric Department 2, Rabat Children’s Hospital, BP 6527 Rabat, Morocco; Human Molecular Genetics Laboratory, NIBGE-PIEAS, Faisalabad 61010, Pakistan; Department of Biological and Biomedical Sciences, The Aga Khan University, Karachi, Karachi City, Sindh 74800, Pakistan; Department of Paediatric Gastroenterology, Hepatology and Genetic Diseases, Children’s Hospital, University of Child Health Sciences, Lahore, Punjab 54000, Pakistan; Department of Paediatric Gastroenterology, Hepatology and Genetic Diseases, Children’s Hospital, University of Child Health Sciences, Lahore, Punjab 54000, Pakistan; Service de Génétique, CHU (Centre Hospitalier Universitaire) de La Réunion, Reunion Island, 97400 Saint-Denis, France; Service de Génétique, CHU (Centre Hospitalier Universitaire) de La Réunion, Reunion Island, 97400 Saint-Denis, France; Wellcome Centre for Mitochondrial Research, Translational and Clinical Research Institute, Faculty of Medical Sciences, Newcastle University, Newcastle upon Tyne NE2 4HH, UK; NHS Highly Specialised Service for Rare Mitochondrial Disorders, Newcastle upon Tyne Hospitals NHS Foundation Trust, Newcastle upon Tyne NE2 4HH, UK; Medical Genetics Department, School of Medicine (KUSoM), Koç University, Istanbul 34450, Turkey; Technical University of Munich, Faculty of Medicine, Chair of Social Pediatrics, Heiglhofstr. 65, 81377 Munich, Germany; Prenatal Medicine Munich, Lachnerstrasse 20, Munich 80639, Germany; Department of Clinical and Movement Neurosciences, UCL Queen Square Institute of Neurology, University College London, London WC1N 3BG, UK; Department of Pediatric Rehabilitation, University Hospital Northern Norway, Tromsø 9019, Norway; Department of Medical Genetics, University Hospital of North Norway, Tromsø 9038, Norway; Department of Clinical and Experimental Medicine, University of Messina, Messina 98122, Italy; Genetics Department, Hospices Civils de Lyon, Lyon 69002, France; Genetics Department, Hospices Civils de Lyon, Lyon 69002, France; Genetics Department, Hospices Civils de Lyon, Lyon 69002, France; GENDEV Team, CRNL, INSERM U1028, CNRS UMR 5292, UCBL1, Lyon 69675, France; Department of Zoology, Abbottabad University of Science and Technology, KP 22500, Pakistan; Department of Zoology, Abbottabad University of Science and Technology, KP 22500, Pakistan; Sheffield Institute for Translational Neuroscience, The University of Sheffield, Sheffield S10 2HQ, UK; Technopole, Milan 20157, Italy; Department of MLT, Abbottabad University of Science and Technology KP, Abbottabad 22500, Pakistan; Department of Zoology, Abbottabad University of Science and Technology KP, Abbottabad 22500, Pakistan; Department of Neuromuscular diseases, UCL Queen Square Institute of Neurology, London WC1N 3BG, UK; Genomics England, London E14 5AB, UK; Children’s Medical Center, Pediatrics Center of Excellence, Tehran University of Medical Sciences, Tehran 14197 33151, Iran; 3billion inc, 416 Teheran-ro, Gangnam-gu, Seoul, Republic of Korea; Department of Genomic Insights, Centogene GmbH, Rostock 18055, Germany; Medical Genetics Department, School of Medicine (KUSoM), Koç University, Istanbul 34450, Turkey; Department of Clinical and Experimental Medicine, University of Messina, Messina 98122, Italy; Department of Pediatrics, Boston Children’s Hospital and Harvard Medical School, Boston, MA 02115, USA; School of Biological Sciences, University of the Punjab, Lahore 54590, Pakistan; Department of Developmental-Behavioral Pediatrics, The Children’s Hospital, University of Child Health Sciences (UCHS-CH), Lahore 54600, Pakistan; Manchester Centre for Genomic Medicine, Manchester University NHS Foundation Trust, Manchester M13 9WL, UK; Manchester Centre for Genomic Medicine, Manchester University NHS Foundation Trust, Manchester M13 9WL, UK; Manchester Centre for Genomic Medicine, Manchester University NHS Foundation Trust, Manchester M13 9WL, UK; Division of Evolution, Infection and Genomics, School of Biological Sciences, Faculty of Biology, Medicine and Health, University of Manchester, Manchester M13 9PL, UK; Manchester Centre for Genomic Medicine, Manchester University NHS Foundation Trust, Manchester M13 9WL, UK; Division of Evolution, Infection and Genomics, School of Biological Sciences, Faculty of Biology, Medicine and Health, University of Manchester, Manchester M13 9PL, UK; Department of Neurosciences, University of California, San Diego, La Jolla, CA 92093, USA; Rady Children’s Institute for Genomic Medicine, San Diego, CA 92123, USA; NIHR Oxford Biomedical Research Centre, Centre for Human Genetics, University of Oxford, Oxford OX3 7BN, UK; Department of Neuromuscular diseases, UCL Queen Square Institute of Neurology, London WC1N 3BG, UK; Department of Biological and Biomedical Sciences, The Aga Khan University, Karachi, Karachi City, Sindh 74800, Pakistan; Faculty of Life Sciences, Health Services Academy, Islamabad 44000, Pakistan; UO Neuroradiologia, IRCCS Istituto Giannina Gaslini, 16147 Genoa, Italy; Department of Clinical and Experimental Epilepsy, UCL Queen Square Institute of Neurology, London WC1N 3BG, UK; Department of Neuromuscular diseases, UCL Queen Square Institute of Neurology, London WC1N 3BG, UK

**Keywords:** RBL2, cell cycle, neurodevelopmental disorder, *Drosophila*, Rbf

## Abstract

Retinoblastoma (RB) proteins are highly conserved transcriptional regulators that play important roles during development by regulating cell-cycle gene expression. RBL2 dysfunction has been linked to a severe neurodevelopmental disorder. However, to date, clinical features have been described in only six individuals carrying five biallelic predicted loss-of-function (pLOF) variants.

To define the phenotypic effects of *RBL2* mutations in detail, we identified and clinically characterized a cohort of 35 patients from 20 families carrying pLOF variants in *RBL2*, including 15 new variants that substantially broaden the molecular spectrum. The clinical presentation of affected individuals is characterized by a range of neurological and developmental abnormalities. Global developmental delay and intellectual disability were observed uniformly, ranging from moderate to profound and involving lack of acquisition of key motor and speech milestones in most patients. Disrupted sleep was also evident in some patients. Frequent features included postnatal microcephaly, infantile hypotonia, aggressive behaviour, stereotypic movements, seizures and non-specific dysmorphic features. Neuroimaging features included cerebral atrophy, white matter volume loss, corpus callosum hypoplasia and cerebellar atrophy.

In parallel, we used the fruit fly, *Drosophila melanogaster*, to investigate how disruption of the conserved RBL2 orthologue Rbf impacts nervous system function and development. We found that *Drosophila Rbf* LOF mutants recapitulate several features of patients harbouring *RBL2* variants, including developmental delay, alterations in head and brain morphology, locomotor defects and perturbed sleep. Surprisingly, in addition to its known role in controlling tissue growth during development, we found that continued *Rbf* expression is also required in fully differentiated post-mitotic neurons for normal locomotion in *Drosophila*, and that adult-stage neuronal re-expression of *Rbf* is sufficient to rescue *Rbf* mutant locomotor defects.

Taken together, our study provides a clinical and experimental basis to understand genotype–phenotype correlations in an *RBL2*-linked neurodevelopmental disorder and suggests that restoring *RBL2* expression through gene therapy approaches might ameliorate some symptoms caused by *RBL2* pLOF.

## Teaser

RBL2 dysfunction, which disrupts cell-cycle gene expression, has been linked to a severe neurodevelopmental disorder. Aughey *et al.* characterize a cohort of patients carrying loss-of-function mutations in *RBL2*, identifying novel molecular variants and clinical features, and gaining mechanistic insights through a fruit fly model.

## Introduction

Retinoblastoma (RB) proteins play well-defined roles in regulating cell-cycle gene expression during development.^1^ The mammalian RB family consists of three members (RB1, RBL1 and RBL2), which share overlapping functions alongside specific roles. RB proteins antagonize the action of E2F transcription factors, which can result in the activation or repression of gene expression depending on genomic context. Mutations impacting the function of RB proteins are linked to an array of disease states. For example, RB1 is a well-known tumour suppressor, with loss-of-function (LOF) mutations associated with several types of neoplastic lesions, including retinoblastoma, prostate cancer, breast cancer, lung cancer and osteosarcoma.^2-7^ RBL1 also acts as a tumour suppressor by inhibiting E2F1 and other E2F transcription factors, preventing inappropriate progression of cells through the cell cycle; while RBL2 functions as a key regulator of cell division, through interactions with E2F4 and E2F5, and promotes senescence by repressing repair genes, controlling DNA methylation and influencing telomere length.^8-10^

Interestingly, RBL1 and RBL2 have been found to regulate neuronal differentiation and the survival of post-mitotic neurons.^11^ Correspondingly, pathogenic variants in *RBL2* have been associated with severe developmental delay, dysmorphic features, microcephaly, seizures and behavioural abnormalities.^12-14^ However, clinical features associated with *RBL2* pathogenic variants have been characterized in only a limited number of individuals, precluding a comprehensive characterization of this disorder. Furthermore, the cell types in which *RBL2* expression is required to promote neural development and function remain unclear.

To define the phenotypic effects of *RBL2* mutations in detail, we identified and clinically characterized a cohort of 35 patients from 20 families carrying homozygous or compound heterozygous predicted LOF (pLOF) variants in *RBL2*. These studies have expanded the clinical spectrum and identified the most common dysmorphic and neuroradiological features linked to the disorder. Additionally, we have broadened the molecular spectrum by identifying 15 new disease-causing variants, providing additional support for *RBL2* LOF as basis of this disorder.

*RBL2* null mice display embryonic lethality coupled with impaired neurogenesis and enhanced apoptosis.^15^ Therefore, we used the fruit fly, *Drosophila melanogaster*, to investigate how disruption of the conserved RBL2 orthologue Retinoblastoma-family protein (Rbf) impacts nervous system function and development. We found that *Drosophila Rbf* hypomorphs recapitulate several developmental features of patients harbouring *RBL2* variants. Surprisingly, in addition to its known role in controlling tissue growth during development, we found that continued Rbf expression is also required in fully differentiated post-mitotic neurons for normal locomotion in *Drosophila* and that adult-stage neuron-specific re-expression of Rbf is sufficient to rescue *Rbf* mutant locomotor defects.

Collectively, our work substantially broadens the clinical characterization of *RBL2*-linked neurodevelopmental disorder and suggests that RBL2 plays critical neurological roles both in dividing neural precursors and in differentiated post-mitotic neurons.

## Materials and methods

### Patient identification and genetic investigation

#### Patient recruitment

The affected individuals were identified through data sharing with collaborators and screening databases of several diagnostic and research genetic laboratories worldwide, in addition to using GeneMatcher.^16^ Patient consent was obtained according to the Declaration of Helsinki. Informed consent forms allowing for participation were signed by all study participants and/or their parents or guardians, and patient studies were approved by ethical committees within the institutions in which the studies were performed. Genome/exome sequencing was performed on genomic DNA extracted from blood in different diagnostic or research laboratories worldwide, and if required, candidate variants were confirmed by Sanger sequencing in the available samples from other members of the families.

#### Ethical declarations

Individuals and/or their legal guardians recruited for this study gave informed consent for their participation. This study received approval from the Review Boards and Bioethics Committees at University College London Hospital (project 06/N076). Permission for inclusion of their anonymized medical data in this cohort, including photographs, was obtained using standard forms at each local site by the responsible referring physicians.

#### Clinical assessment

Detailed clinical data and family history were collected for new and reported cases in the form of completing a clinical proforma by the recruiting clinicians. Brain MRIs were reviewed by an experienced paediatric neuroradiologist (M.S.). Video segments of seven patients were suitable for fine analysis of the stereotypies by an experienced neurologist (E.F.). Facial photographs and/or videos were reviewed for 28 patients from 16 families, including 22 new patients from 12 families and six previously published patients from four families.^12-14^ Their dysmorphic features were described based on the terminology recommended by Elements of Morphology.^17^ Where no term was available for a dysmorphic feature seen in a patient, Human Phenotype Ontology (HPO) terminology was used instead.^18^

#### RT-PCR and RNA-sequencing

RNA was extracted from 1 ml PAXgene blood aliquots using the Qiagen blood RNA kit and a QIAcube Classic (QIAgen). Complementary DNA synthesis and RT reactions were performed either with QuantiTect reagents (Families F1 and F7) or using Applied Biosystems high-capacity complementary DNA reverse transcription kit with RNase inhibitor (Family F16). For Family F7, PCR amplification used primers in exon 6 (TGGCCTAGTTTTGGAAGCAA) and exon 9 (CACTTGGTGCATTCCTGAGG), and Sanger sequencing was performed using BigDye chemistry on an ABI 3730XL. For Family F16, PCR amplification of exons 15–20 was performed using primers spanning the exon 15 and 16 boundary (TTCCTGTGCAAGGTATTGCC) and exon 20 (CTGTGAGGCGAGTAGGTGTG).

Library preparation used TruSeq stranded total RNA with globin depletion on 100–200 ng input. Sequencing on the NovaSeq used 76 bp paired-end reads, with a minimum of 50 million reads per sample. Alignment to GRCh38 used STAR7 (v.2.7.3a with the -twopassMode Basic option). and the resulting BAM files were sorted/indexed with Samtools (v.1.9).^8^ Mapped reads per gene were calculated using bedtools coverage (using the -split option) considering the whole gene region in addition to only exonic or intronic regions according to gencode v.30 gene definitions. Transcripts per million were calculated for each gene using a custom R script. Considering *RBL2*, normalized expression for the whole gene was calculated as gene mapping reads/total million reads. Normalized expression for intronic and exonic regions was obtained by first dividing intronic/exonic mapping reads for total million reads, then normalizing this value for the total fraction of intronic/exonic reads in the sample to account for variability in intron/exon region capture seen across samples.

### *Drosophila* studies

#### *Drosophila* husbandry

All stocks and experimental crosses were raised on standard fly-food media and kept at 25°C with 12 h light/12 h dark cycles. *Drosophila* strains used in this study are listed in Supplementary Table 1. For behavioural experiments, isogenized lines (indicated in Supplementary Table 1) were generated by outcrossing each mutation or transgene insertion into the iso31 strain of *w*^11,18^ for five generations.^19^

#### Immunohistochemistry

Immunohistochemical experiments were performed as previously described.^20^ Briefly, adult or larval brains were dissected in PBS and fixed in 4% paraformaldehyde (MP Biomedicals) for 20 min at room temperature. Tissues were washed with PBST (PBS containing 0.3% Triton X-100), blocked in 1% goat serum in PBST and incubated in primary antibody overnight at 4°C. Following primary antibody incubation, tissues were washed a further three times in PBST and incubated overnight in secondary antibody. Antibodies used in this study included mouse anti-ELAV (Developmental Studies Hybridoma Bank, Elav-9F8A9),^21^ rabbit anti-cleaved DCP1 (Cell Signaling Technology, Catalogue No. 9578) and mouse anti-Repo (Developmental Studies Hybridoma Bank, 8D12).^22^ Images were processed using Fiji.^23^ For measurement of morphological attributes (e.g. optic lobe areas), regions of interest were defined from maximum-intensity projections using the freehand selection tool before measuring dimensions of the selected areas. Only brains with no detectable damage following the dissection and mounting procedure were included for analysis.

#### *Drosophila* behavioural analyses

*Drosophila* activity was assayed using single or multibeam *Drosophila* Activity Monitor systems (DAM; Trikinetics) as previously described.^24,25^ Briefly, individual flies obtained between 3 and 5 days after eclosing were loaded into glass tubes containing 4% sucrose and 2% agar (w/v) and sealed with cotton-wool plugs. For locomotor activity and sleep measurements, monitors were kept at 25°C with 12 h light–12 h dark cycles for 2 days to acclimate. On the third day, locomotor activity and sleep were recorded for 24 h. For measurements of peak activity at zeitgeber time (ZT)0–1 or ZT12–13, activity was taken from the hour after lights on or off during the third day. For measurements of the period and strength of free-running circadian patterns of locomotion, activity of adult flies was recorded in constant-dark conditions (DD) over a 5-day period. DAM data were analysed using the Rethomics R package.^26^ For sleep studies, a sleep bout was defined as a 5 min period of inactivity during which no beam breaks were quantified in the DAM system (the common standard in the field).^27^ Only flies surviving for the full 3 days were included for activity/sleep analysis. For adult-specific knockdown and rescue experiments, flies were raised at 18°C until 2 days post-eclosion, at which point they were loaded into DAM monitors and moved to 29°C for 3 days (or remained at 18°C for controls).

Larval locomotion assays were conducted by transferring wandering third instar larvae to a large arena containing 2% agar. The arena was placed into a 25°C incubator, and larvae were left to acclimate for 30 s. Larval crawling was video recorded for 1 min. Video files were analysed using ImageJ to calculate the total distance travelled.

Negative geotaxis (climbing) assays were conducted as previously described.^28^ Briefly, cohorts of 10 flies were transferred to clean glass measuring cylinders and left to acclimate for ≥20 min. Flies were firmly tapped down three to five times, and the number of flies crossing an 8 cm vertical threshold in 12 s was recorded. Three technical replicates were included for each genotype.

### Statistical analyses

Statistical data analysis was performed using R or GraphPad Prism. Datasets were first tested for normality using the Shapiro–Wilk normality test. Statistical analyses were performed using a *t*-test with Welch’s correction or one-way ANOVA with Dunnett’s multiple comparisons *post hoc* test if data were normally distributed and with the Mann–Whitney U-test or Kruskal–Wallace test with Dunn’s multiple correction if data were non-normally distributed.

## Results

### Clinical profile of the study cohort

**Figure 1:**
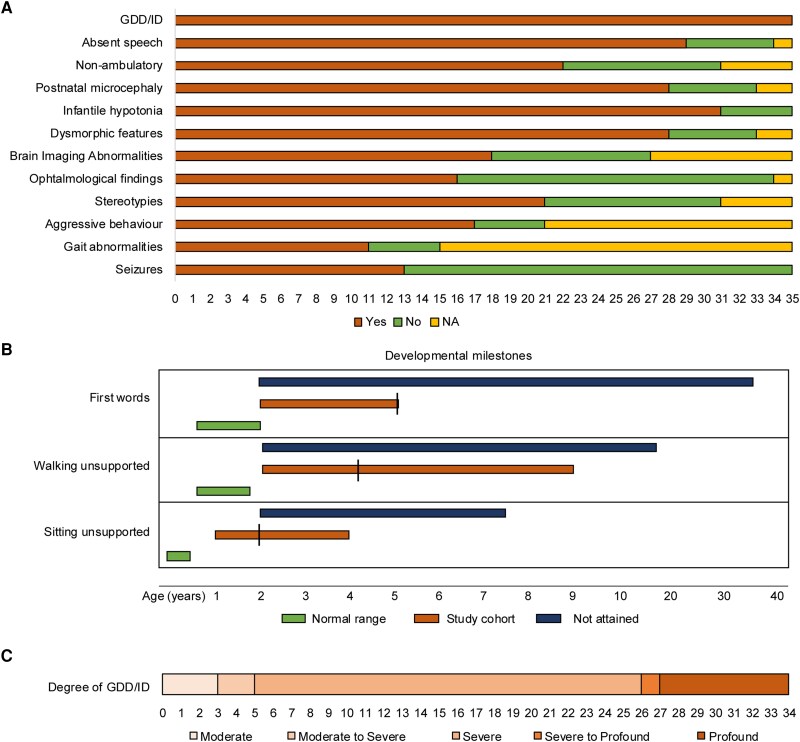
***RBL2-*related disorder is characterized by a range of neurological, behavioural and developmental abnormalities.** (**A**) Representation of the most frequent clinical features observed in the *RBL2* patients (*y*-axis, clinical features; *x*-axis, number of patients). (**B**) Time-line-style schematic diagram outlining the acquisition of key developmental milestones observed in the affected individuals. Most of the individuals did not attain independent sitting, walking or speech development (blue bar indicates range at last evaluation), and the others presented delayed acquisition (orange, line indicates median age). Normal range is indicated in green. (**C**) Schematic depiction of the degree of global developmental delay/intellectual disability (GDD/ID) observed in the patients (number of patients indicated at *bottom*). The spectrum ranged from moderate (*left*) to profound (*right*).

The overall cohort comprised 17 females and 18 males, whose age at last evaluation ranged between 2 and 36 years [median 13 years, interquartile range (IQR) 12 years]. An overview of the clinical findings can be found in Fig. 1A and Table 1. Detailed clinical information is available in Supplementary Table 2. Consanguinity was reported in 17 families (85%). Pregnancy and delivery were unremarkable for most the patients for whom information was available (27/31, 87%), and all newborns were at term. Birth parameters of length, weight and head circumference, when available, were within normal ranges for almost all the infants. Only two infants presented with decreased head circumference at birth (HP:0011451), and two had low birth weight (HP:0001518). Most of the newborns (31/35, 89%) manifested infantile hypotonia (HP:0008947). Failure to thrive (HP:0001508) and feeding difficulties in the infantile period were documented in 52% and 30% of those examined, respectively (11/21 and 8/26, respectively).

**Table 1:** Overview of the clinical features observed in the cohort

Family identity	1		2	3		4	5		6	7	8		9			10	11		12		13	14	15					16		Lit.	Total
Patient	1	2	3	4	5	6	7	8	9	10	11	12	13	14	15	16	17	18	19	20	21	22	23	24	25	26	27	28	29	30–35	35
Sex	F	M	F	F	F	F	M	M	M	F	M	M	M	M	M	M	F	F	F	M	F	M	M	F	F	M	F	F	M	3F, 3M	17F, 18M
Age, years	17	16	13	18	14	2	6	3	4	13	21	29	18	14	3	7	17	9	2	9	10	3.75	26	16	22	21	10	5	1.5	6–36	2–36
Microcephaly	+	+	+	+	+	+	+	+	+	+	−	−	+	+	+	N	+	+	+	+	+	−	N	+	+	+	+	+	+	4/6	28/33
Non-mobile	+	+	−	+	+	+	+	+	+	−	−	−	−	+	+	+	+	+	+	N	+	+	−	−	−	−	−	+	+	4/5	22/31
Non-verbal	+	+	−	+	+	+	+	+	N	+	−	−	+	+	+	+	+	+	+	+	+	+	+	+	+	+	+	+	+	4/6	29/34
GDD/ID	+	+	+	+	+	+	+	+	N	+	+	+	+	+	+	+	+	+	+	+	+	+	+	+	+	+	+	+	+	6/6	34/34
Moderate	−	−	−	−	−	+	+	+	N	−	−	−	−	−	−	−	−	−	−	−	−	−	−	−	−	−	−	−	−	−	3/34
Severe	+	+	−	−	−	−	−	−	N	+	+	+	+	+	+	+	−	−	+	+	+	−	+	+	+	+	+	−	+	5/6	23/34
Profound	−	−	+	+	+	−	−	−	N	−	−	−	−	−	−	−	+	+	−	−	−	+	−	−	−	−	−	+	−	1/6	8/34
Hypotonia	+	−	+	+	+	+	−	−	N	+	−	−	−	−	−	+	−	−	+	+	−	+	N	−	−	−	−	+	+	1/1	13/28
Hypertonia	−	+	−	−	−	−	−	−	N	−	−	−	−	+	+	−	+	+	−	−	+	−	N	−	−	+	−	−	−	− (1)	7/28
Dystonia	−	+	−	+	+	−	−	−	+	−	−	−	−	−	−	N	−	−	+	+	−	+	N	−	−	−	−	+	−	− (1)	8/28
Tremor	−	+	+	+	+	+	−	−	−	−	−	−	−	−	−	N	−	−	−	−	+	−	N	−	−	−	−	−	−	− (1)	6/28
Behavioural problems	−	+	+	+	+	+	+	+	−	+	−	+	+	+	+	+	+	+	−	+	+	+	+	+	+	+	+	+	+	6/6	31/35
Stereotypy	−	+	+	+	+	+	−	−	−	+	−	−	−	−	−	+	+	+	−	+	+	+	N	+	+	+	+	+	+	3/4	21/31
Sleep issues	N	+	N	+	N	N	N	N	N	N	−	−	N	N	N	N	N	N	+	+	N	N	N	+	+	+	+	−	+	3/3	12/15
Seizures	−	−	+	+	+	−	−	+	−	+	−	−	−	−	−	−	−	−	+	+	−	−	−	−	−	+	−	+	+	3/6	13/35
Brain anomaly	+	+	+	+	+	+	N	+	−	+	−	+	N	N	N	+	−	−	+	−	−	+	N	N	N	−	N	+	+	4/5	18/27
Eye issues	+	−	−	+	+	+	+	+	+	+	−	−	−	−	−	−	−	−	−	−	+	N	−	−	−	−	−	+	+	5/6	16/34

F = female; GDD/ID = global developmental delay/intellectual disability; Lit. = patients described in existing literature; M = male; N = not measured.

Global developmental delay (HP:0001263) and intellectual disability (HP:0001249) were reported in all the affected individuals (35/35, 100%). All patients (35/35, 100%) presented motor delay (HP:0001270), and most of the affected children presented a delay in achieving unsupported sitting (22/30 delayed, 3/30 not attained, median 2 years, IQR 1.25 years). The majority never attained independent walking (21/34, 62%), and the remainder had delayed acquisition (median 4 years, IQR 1.95 years). In the same way, most children (25/34, 74%) showed complete lack of development of speech and expressive language abilities (HP:0001344), while in the remaining individuals (9/34, 26%) development of speech was delayed (HP:0000750) and involved the use of only a few words (median 5 years, IQR 1.5 years) (Fig. 1B). Regression of motor and cognitive abilities (HP:0002376) was reported in five patients (5/29, 17%). The degree of global developmental delay/intellectual disability, whether assessed through formal testing or based on clinical judgment, ranged from moderate (3/34) to severe (23/34) and profound (8/34) (Fig. 1C). Behavioural abnormalities (31/35, 89%) included stereotypies (21/31, 68%), aggressive behaviour (17/21, 81%) and autistic features (9/25, 36%). When information on sleep was available, sleep difficulties (HP:0002360) were documented in 12/15 (80%) individuals.

Video segments of seven patients were suitable for fine analysis of the stereotypies. The stereotypies usually involved the cervicofacial area (head and/or orofacial region) along with the distal part of the upper limbs, typically in the form of hand clasping/squeezing and mouthing, and finger wringing (Supplementary Video 1). Movement abnormalities included dystonia (HP:0001332) (8/27, 30%) and tremor (HP:0001337) (6/27, 22%).

Seizures occurred in 37% of the individuals (13/35). Age at onset of the seizures ranged from 1 to 20 years (median age 6 years, IQR 9 years). According to the International League Against Epilepsy (ILAE) classification, all patients presented a generalized seizure onset (HP:0002197) (9/9), while two patients also presented a focal onset (HP:0007359) (2/9). In four patients, seizures onset was not specified. Seizures were classified as motor in all the patients and were either tonic–clonic (*n* = 6) or myoclonic (*n* = 2). Seizure duration varied from 1 to 10 min. Clustering was reported in two of five patients. Febrile seizures were documented in four patients. Most patients were well controlled with valproate (*n* = 5), levetiracetam (*n* = 1) or a combination of both (*n* = 2). Two patients presented with intractable seizures. EEG abnormalities (4/5, 80%) included focal, multifocal and diffuse epileptiform discharges, slowing of background activity and subcortical changes. EEG was performed in seven patients with no evident clinical seizures and documented epileptogenic discharges in two of them.

Neurological examination showed increased tendon reflexes (HP:0001347) (11/23, 48%), muscle weakness (HP:0001324) (13/27, 48%), axial hypotonia (HP:0008936) (12/27, 44%) and spasticity (HP:0001257) (11/31, 35%). Ophthalmological evaluation revealed the presence of abnormal findings in almost half of the cases (16/34, 47%), including strabismus (HP:0000486) (9/21, 43%), nystagmus (HP:0000639) (7/28, 25%), refractive defects (4/27,15%), poor vision (8/33, 24%), optic disc anomalies (4/24, 17%) and orbital mass (2/33, 6%).

**Figure 2:**
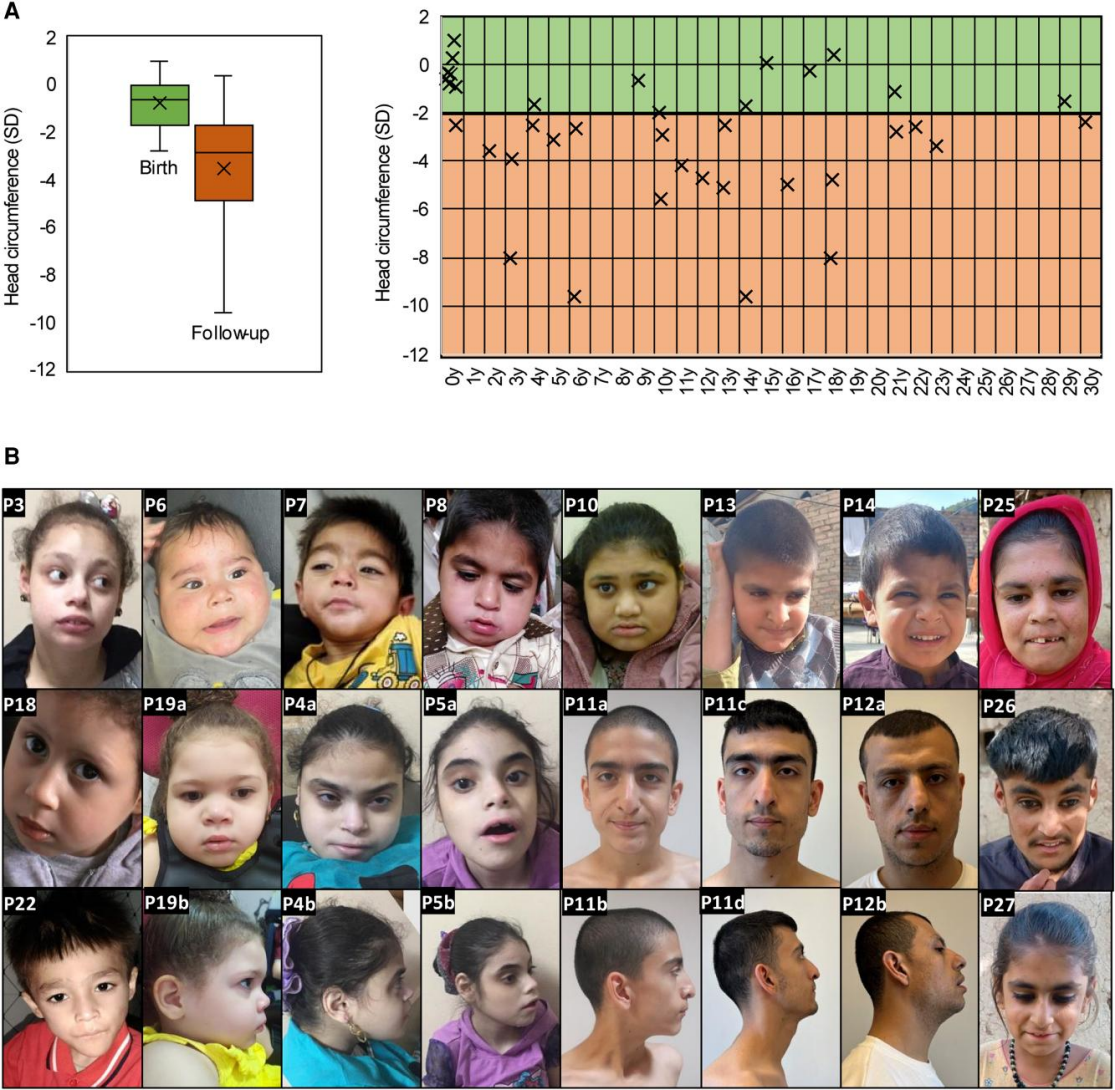
***RBL2* patients present postnatal microcephaly and dysmorphic features, without a recognizable facial ‘gestalt’.** (**A**) *Left*: Box plot showing the range of head circumference measurements in *RBL2* predicted loss-of-function patients, expressed in standard deviations (SDs) from the mean of healthy controls. The box delineates the range between first and third quartile, the cross (×) represents the mean, and the line that divides the box indicates the median of the whole cohort. Head circumference was within normal ranges at birth and reduced at last examination. *Right*: Head circumference measurements with age at last follow-up across individual patients. (**B**) Facial features of the patients. P = patient.

At the last evaluation, 85% of the patients (28/33) were microcephalic (HP:0000252) (Fig. 2A). Dysmorphic features were described in 90% of the cases and included, based on photographic assessment, low anterior hairline (50%), narrow forehead/bifrontal/bitemporal narrowing (83.3%), full or broad nasal tip (77.8%), thick/full lower lip vermilion (66.7%) and broad or tall pointed chin (77.8%) (Fig. 2B and Supplementary Table 3). When available, metabolic testing was normal for almost all the patients (17/20, 85%). Interestingly, repeated very long-chain fatty acid testing for two siblings showed elevated C26 with a normal C26 ratio, and one patient presented hyperlactacidaemia.

### Neuroradiological features of *RBL2* patients

**Figure 3:**
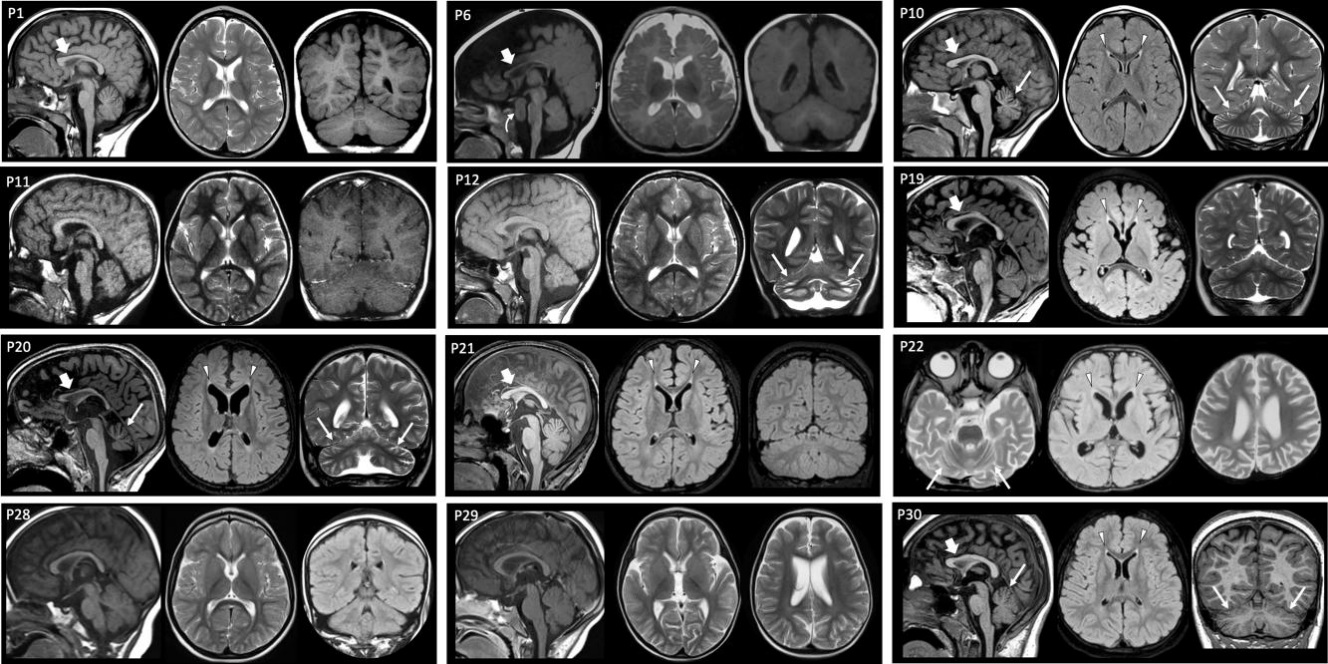
**Neuroimaging features of RBL2-related disorder.** Sagittal T_1_-weighted image (*left*), axial T_2_-weighted or FLAIR image (*middle*) and coronal T_1_- or T_2_-weighted or FLAIR image (*right*). Most subjects have an enlargement of the cerebral CSF spaces, with an anteroposterior gradient associated with thinning of the corpus callosum (thick arrows), particularly in the anterior portions. There is additional cerebellar atrophy in P10, P12, P20, P22 and P30 (thin arrows). Bilateral mild-to-moderate signal changes are noted at the level of the forceps minor in P10, P19, P20, P21, P22 and P30 (arrowheads). Note the large prepontine lesion in P6 (curved arrow). FLAIR = fluid-attenuated inversion recovery; P = patient.

Brain MRIs were available for review in 15/35 cases (mean age at MRI 7 years, range 8 months to 17 years). The most frequent neuroimaging finding was a mild-to-moderate decrease in cerebral volume, suggesting cerebral atrophy with an anteroposterior gradient, and thin corpus callosum (11/15, 73.3%) (Fig. 3). Reduced white matter volume with ventricular enlargement was associated in 9/15 cases. In 9/15 subjects (60%), we found white matter signal abnormalities, including faint to marked focal signal changes at the level of forceps minor (8/9), delayed myelination (2/9) and multiple patchy frontal signal changes (1/9). Mild-to-moderate cerebellar atrophy was noted in 7/15 individuals (46.6%), with dentate signal changes in three cases and clear progression in one subject with a follow-up MRI; in one individual, there were also bilateral widespread subcortical signal changes. In four other subjects (26.6%) there was hypoplasia of the inferior portion of the cerebellar hemispheres and/or vermis, with associated foliar anomalies in one case. Optic nerve thinning was detected in 5/15 (33.3%) individuals. Calcifications in the basal ganglia were found in 2/15 (13.3%) cases. Finally, expansile lesions were found in two subjects: a large mass extending from the third ventricular floor to the prepontine cisterns (hypothalamic hamartoma versus ectopic cerebellar tissue) in P6 and a cystic mandibular lesion in P30 (Supplementary Table 4).

### Molecular spectrum of *RBL2* variants

**Figure 4:**
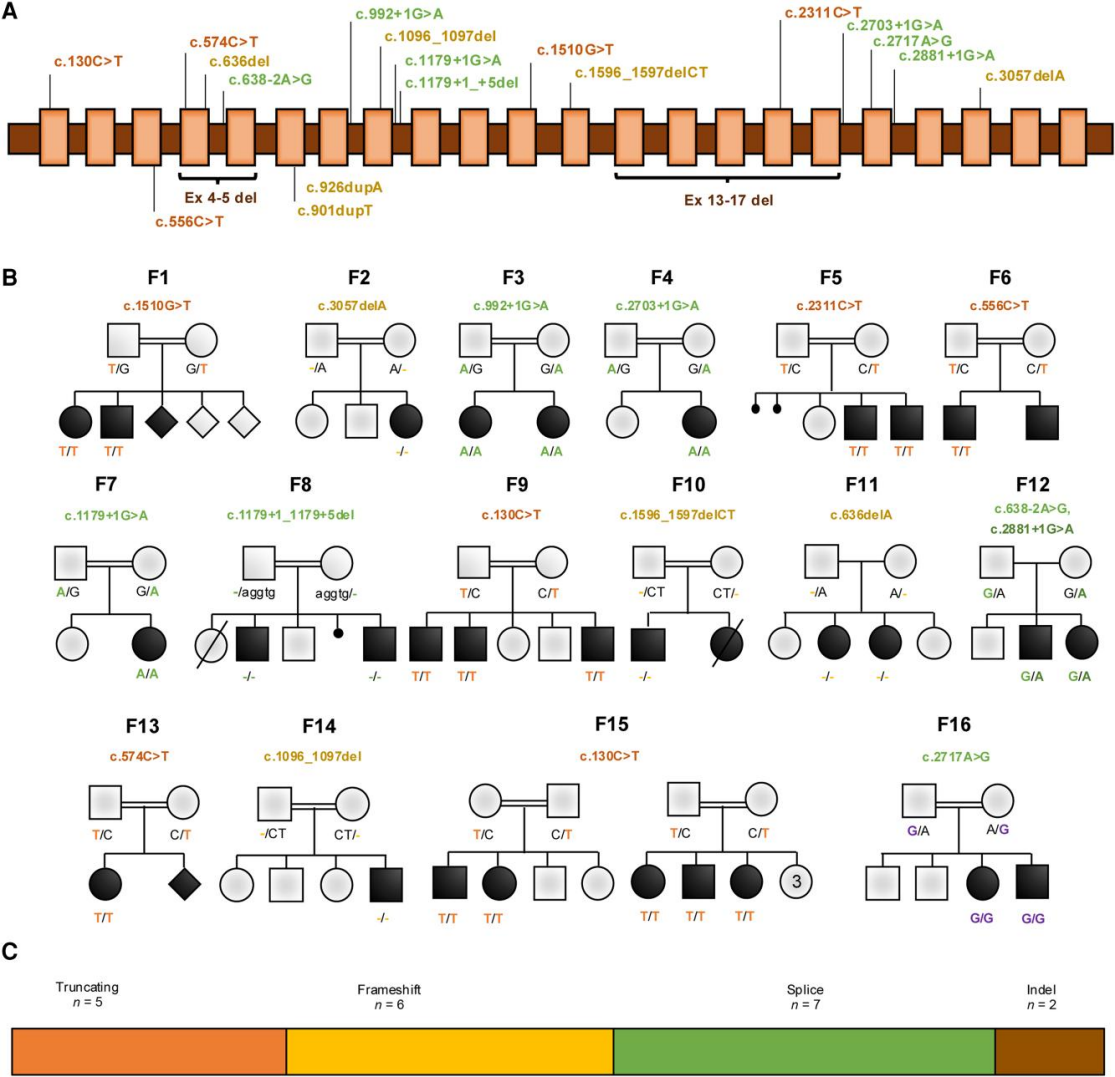
**Molecular spectrum of loss-of-function variants in *RBL2.*** (**A**) Schematic representation of the location of variants on the *RBL2* gene. *Top*: Newly reported variants. *Bottom*: Previously reported variants. (**B**) Pedigrees of the newly reported patients. Filled black symbols = affected. Genotype, where indicated, represents the results of segregation. (**C**) Classification of variants according to type.

A total of 20 *RBL2* variants are included in this study (Fig. 4A), 15 of which are newly reported variants not described in the literature. Within the cohort of newly reported families (Fig. 4B), only one affected family carried a previously reported variant (c.556C>T, p.Arg186Ter). Molecular findings are shown schematically in Fig. 4 and described in detail in Supplementary Table 5. The variants were inherited from unaffected heterozygous parents: 31 patients inherited the variant in the homozygous state and four in compound heterozygous state. All variants were either absent or found at very low allele frequencies in multiple variant frequency databases (range 0.0–0.00002). The molecular spectrum hereby described includes nonsense (*n* = 5), frameshift (*n* = 6) splice (*n* = 7) and large deletions (*n* = 2) (Fig. 4C). According to the American College of Medical Genetics (ACMG) classification, six were classified as pathogenic, 13 as likely pathogenic and one as a variant of uncertain significance. All identified variants were predicted to be damaging across a suite of *in silico* tools and are expected to lead to LOF of the protein.

By chance, the proband from Family F1, carrying the truncating mutation c.1510G>T, had previously been entered into a pilot RNA-sequencing study involving 29 unrelated subjects from the 100 000 Genomes Project (100kGP) with a suspected but as yet unsolved genetic disorder. Although c.1510G>T was convincingly validated (Supplementary Fig. 1A), expression analysis revealed a gene normalized expression of 264.7, similar to the median value of 260.5 observed across the cohort. This indicated that c.1510G>T does not lead to nonsense-mediated decay (NMD), therefore suggesting the presence of a truncated protein. Nevertheless, premature termination of translation 44% through the coding sequence (codon 504/1140) would be highly likely to result in a non-functional protein. Intriguingly, we also observed an increased proportion of reads mapping to intronic regions (Supplementary Fig. 1B). These observations could result from altered mRNA processing in transcript molecules carrying the mutation, although additional data are needed to confirm this hypothesis.

Seven of the variants reported here involved consensus splice donor/acceptor sites, and all had SpliceAI delta scores of >0.9. Using SpliceAI-visual,^29^ we determined that for four of these variants exon skipping was the most likely outcome (see https://genome.ucsc.edu/s/AlistairP/RBL2_splice_v3). In Family F7, RT-PCR and Sanger sequencing confirmed that the *in silico* prediction for c.1179+1G>A to result in a 41 bp extension of exon 8 was correct (Supplementary Fig. 1C). For the variant c.2717A>G in Family F16, RT-PCR and Sanger sequencing confirmed the SpliceAI-predicted skipping of exon 18 (Supplementary Fig. 1D, E). Although RNA samples from other families were not available, we note that the prediction for c.1179+1_1179+5del in Family F8 involved upregulation of the same cryptic intronic donor site. The final splice variant c.2703+1G>A in Family F4 was predicted to result in intron retention. These data reveal additional molecular pathways through which mutations in the RBL2 cohort could cause LOF.

### A *Drosophila* model of RBL2-linked pathology recapitulates morphological patient phenotypes

The *D. melanogaster* genome encodes two Rb proteins: Rbf and Rbf2. Of these, Rbf shares the greater similarity to RBL2 (39% similarity and 25% identity; in comparison to 35% similarity and 20% identity for Rbf2). Indeed, 14 distinct databases of orthology relationships place Rbf as the closest *Drosophila* orthologue of RBL2, and RBL2 was the closest match for Rbf in a reverse orthology search (https://flybase.org/reports/FBgn0015799#orthologs). Similarly to RBL2, prior work has shown that *Drosophila* Rbf interacts with and negatively regulates E2F transcription factor activity to repress cell-cycle gene expression.^30-32^ Thus, human RBL2 and *Drosophila* Rbf exhibit both functional and amino-acid conservation. Furthermore, published single-cell RNA-sequencing data indicate that *Rbf* is widely expressed throughout the *Drosophila* nervous system, whereas *Rbf2* is not (Supplementary Fig. 2A and B).^33,34^ Hence, we investigated how loss of Rbf function impacted neural development and behaviour in *Drosophila*.

**Figure 5:**
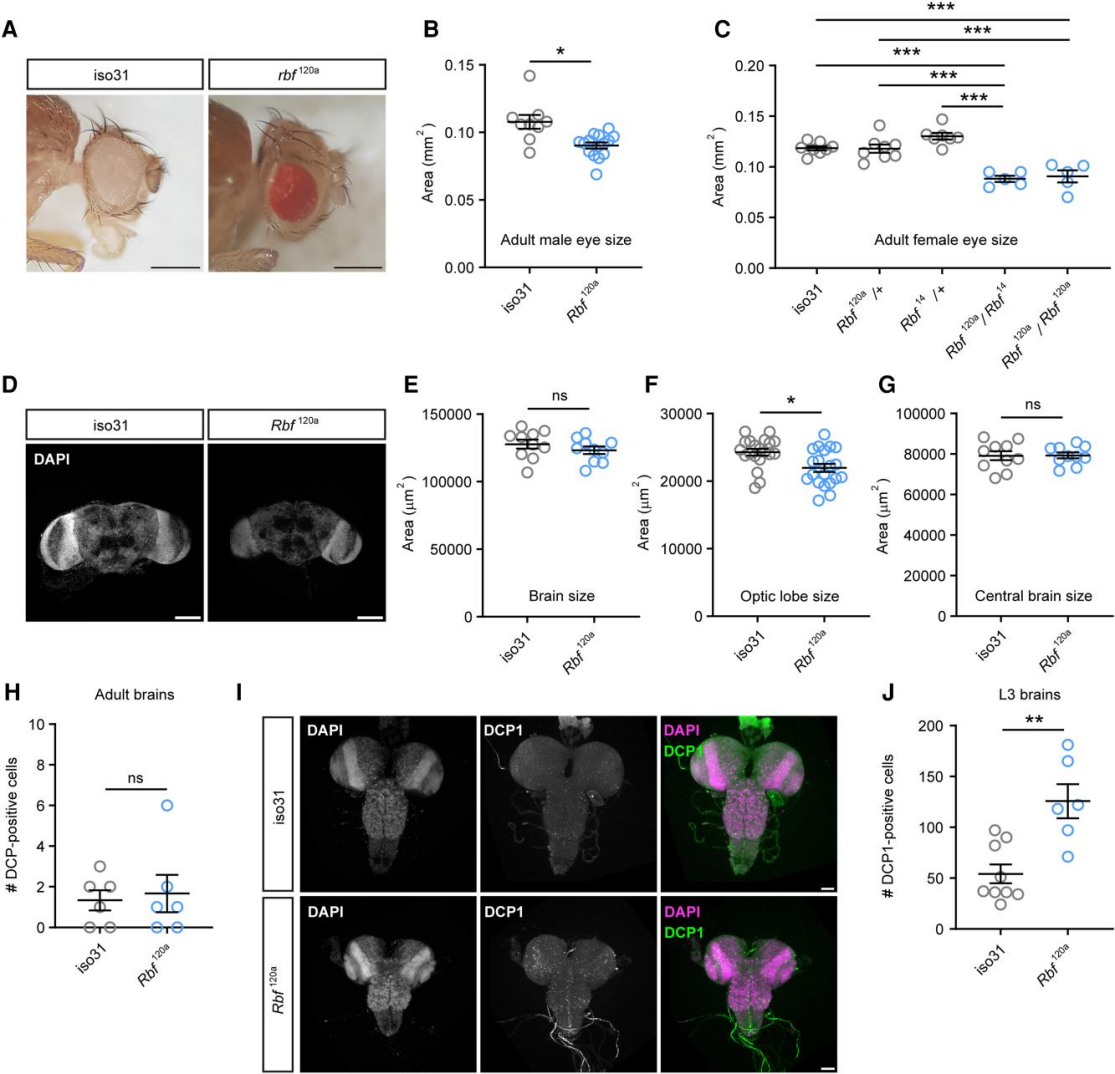
***Drosophila* Rbf regulates head and brain morphology.** (**A**) Representative images of adult eyes in control (iso31) and *Rbf^120a^* hypomorphs. Scale bars: 0.3 mm. (**B**) Quantification of eye sizes in male *Rbf^120a^* hemizygotes (*n* = 16) compared with controls (*n* = 9). (**C**) Quantification of eye sizes in female *Rbf* allelic combinations (*n* = 5–8). (**D**) Representative images of adult brains in control and *Rbf^120a^* adult males. Scale bars: 50 μm. (**E**–**G**) Measurements of brain morphology in control and *Rbf^120a^* hemizygotes adult males (*n* = 10 brains, 10 central brains and 20 optic lobes per genotype). (**H**) Quantification of apoptotic (DCP1^+^) cells in control and *Rbf^120a^* hemizygote adult male brains (*n* = 6 per genotype). (**I**) Representative images of DCP1-labelled control (*n* = 9) and *Rbf^120a^* hemizygote (*n* = 6) third instar larval nervous system. Nuclei are counterstained with DAPI. Scale bars = 20 μm. (**J**) Quantification of apoptosis in control and *Rbf^120a^* hemizygote third instar larval brains. Error bars are the standard error of the mean. *P < 0.05, **P < 0.005, ***P < 0.0005, ns = *P* > 0.05, unpaired *t*-test with Welch’s correction (**B**, **E** and **F**), one-way ANOVA with Dunnett’s *post hoc* test (**C**) or Mann–Whitney U-test (**G**, **H** and **J**).

We set out to determine the extent to which *Drosophila Rbf* LOF phenotypes resemble *RBL2* patient symptoms. Initially, we examined male and female flies hemizygous or homozygous, respectively, for a hypomorphic­ allele of *Rbf* (*Rbf^120a^*) to determine whether partial loss of Rbf function in flies recapitulated morphological and behavioural phenotypes of *RBL2* patients. Although a previous study suggested that eye morphology in *Rbf^120a^* hemizygotes was relatively normal,^35^ we noticed that the size of the eye was significantly smaller in hemizygous *Rbf^120a^* males compared with control flies, although the highly organized ommatidial structure appeared unaffected (Fig. 5A and B). We also examined eye size in female *Rbf^120a^* homozygotes and females trans-heterozygotous for *Rbf^120a^* and the *Rbf^14^* null allele (note that adult *Rbf^14^* homozygotes are embryonic lethal).^36^ Both *Rbf^120a^* homozygote and *Rbf^120a^/Rbf^14^* trans-heterozygous females also displayed smaller eyes compared with wild-type control and *Rbf^120a^*^/+^ or *Rbf^14^*^/+^ heterozygote flies (Fig. 5C).

Given that microcephaly is a clinical feature of *RBL2* patients, we next examined whether brain size was also reduced in *Drosophila Rbf* mutants (Fig. 5D–G). Although overall brain size was not significantly smaller in *Rbf^120a^* hemizygote males (Fig. 5E), we observed a significant reduction in the size of *Rbf^120a^* hemizygote optic lobes, visual processing centres that contain >60% of all neurons in the fly brain^37^ (Fig. 5F). In contrast, the central brain region of *Rbf^120a^* hemizygotes was unaltered (Fig. 5G).

Rb proteins have been linked to apoptosis in humans^38^ and *Drosophila*,^39^ with *Rbf^120a^* mutants displaying increased apoptosis in the eye imaginal disc (the developmental precursor to the adult eye^35^). We therefore reasoned that decreased brain size in *Rbf* mutants might be driven by an increase in cell death. To determine the amount of apoptosis in the brains of *Rbf^120a^* mutants, we stained tissues with anti-DCP1, which recognizes the cleaved version of a caspase protein involved in apoptotic cell death. Examination of adult *Rbf^120a^* brains indicated minimal apoptosis, as was also observed in control adult brains (Fig. 5H). However, examination of larval brains, in which most neurons are in a more immature state, revealed significantly greater numbers of apoptotic cells in *Rbf^120a^* mutants than controls (Fig. 5I and J). This suggests that neuronal precursors and immature neurons are more sensitive to the induction of apoptosis when Rbf is depleted, in agreement with previous observations of the developing eye.^34^ Consistent with this, we observed that larval brain size was also significantly smaller in *Rbf^120a^* mutants compared with controls (Supplementary Fig. 3A–C). In concert with the microcephaly seen in *RBL2* patients, these data reveal a conserved function of the RBL2 and Rbf proteins in controlling head and brain morphology during development.

### *Drosophila Rbf* mutants display developmental delay, motor defects and impaired sleep

**Figure 6:**
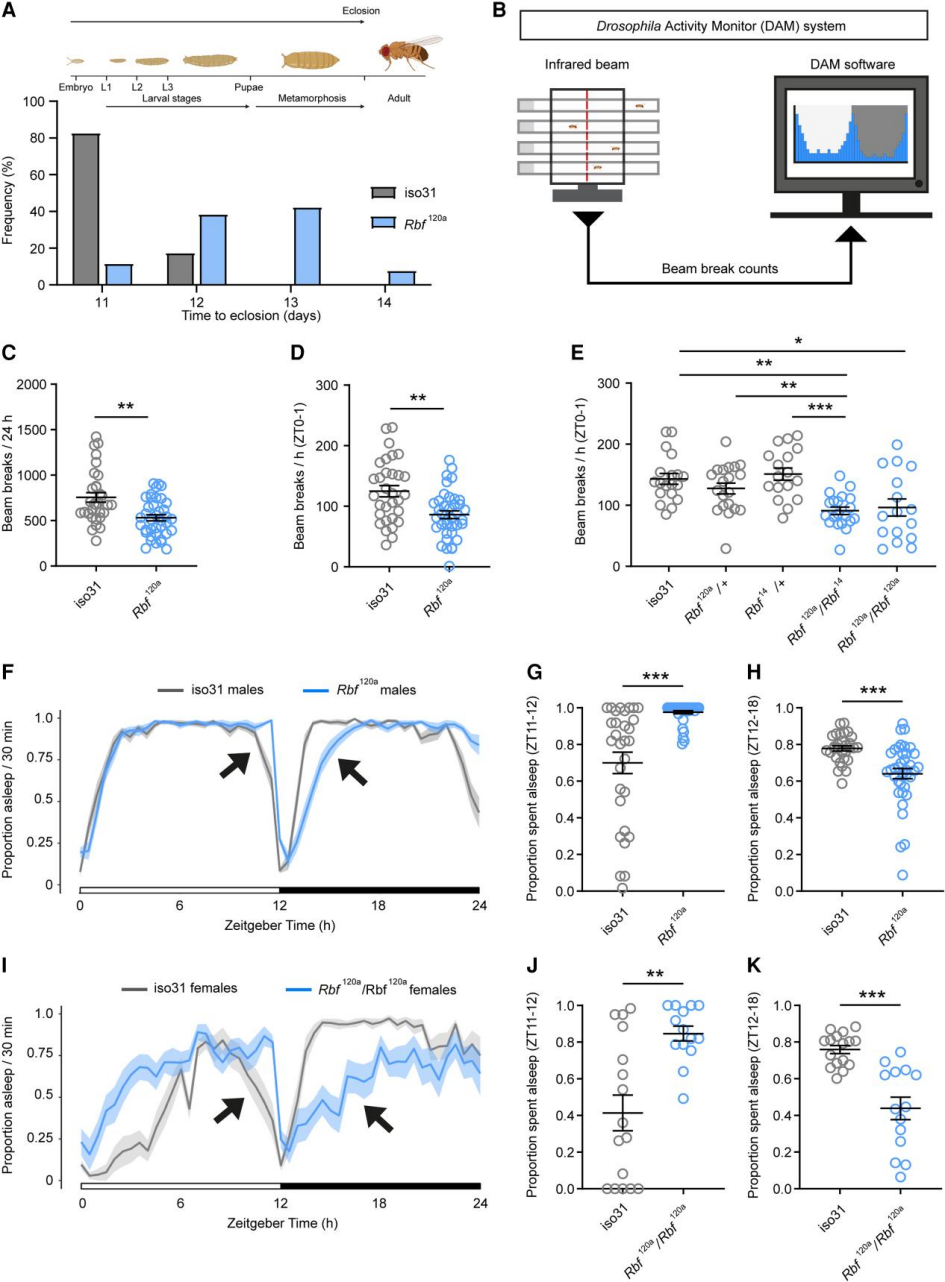
**Loss of Rbf disrupts movement and sleep in *Drosophila*.** (**A**) Schematic diagram illustrating *Drosophila* life cycle and histogram of time to eclosion for iso31 controls (*n* = 201) and *Rbf^120a^* hemizygotes (*n* = 26). (**B**) Schematic representation of the *Drosophila* activity monitor (DAM) system. (**C** and **D**) DAM activity in *Rbf^120a^* hemizygotes (*n* = 38) and controls (iso31; *n* = 31) across a 24 h period (**C**) or during zeitgeber time (ZT) 0–1, a period of peak activity (**D**). (**E**) DAM activity in adult females harbouring trans-heterozygote or heterozygote *Rbf* allelic combinations and in wild-type iso31 controls during ZT0–1. *n* = 16–20. (**F**) Sleep traces of control (iso31) and *Rbf^120a^* hemizygote males showing the proportion of time spent asleep during 30 min windows across a 12 h light/12 h dark period. Loss of evening anticipation (*left arrow*) and reduced sleep during the first half of the night (*right arrow*), in *Rbf^120a^* males. (**G** and **H**) Proportion of time spent asleep during the hour before lights-off (**G**) and the first half of the night (**H**) in control and *Rbf^120a^* males. *n* = 31 iso31 males and 38 *Rbf^120a^* males. (**I–K**) Sleep traces (**I**), proportion of time spent asleep during the hour before lights-off (**J**) and the first half of the night (**K**), in control and *Rbf^120a^* homozygote females. Arrows in **I** again point to loss of evening anticipation (*left arrow*) and reduced sleep during the first half of the night (*right arrow*). *n* = 16 iso31 and 14 *Rbf^120a^* females. Error bars are the standard error of the mean. *P < 0.05, **P < 0.005, ***P < 0.0005, ns = *P* > 0.05, unpaired *t*-test with Welch’s correction (**C** and **J**), Mann–Whitney U-test (**D**, **F**, **G** and **I**) or one-way ANOVA with Dunnett’s *post hoc* test (**E**).

A major component of the *RBL2* patient phenotype is a pronounced delay in reaching developmental milestones. We found that *Drosophila Rbf* mutants also exhibited developmental delay, with a mean of 12.5 days taken from egg laying to eclosion (the emergence of adult flies from the pupal case), compared with 11 days for controls (Fig. 6A and Supplementary Fig. 4A). Given that profound motor delay was observed in all *RBL2* patients, we also tested whether *Drosophila Rbf* mutants exhibited motor defects. To do so, we used the *Drosophila* DAM system,^24^ which quantifies spontaneous activity by recording the number of times that individual flies interrupt an infra-red beam bisecting a glass tube housing each fly (Fig. 6B). *Rbf^120a^* hemizygotes showed significantly lower locomotor activity compared with controls both over a 12 h light–12 h dark period (Fig. 6C) and during a 1 h window following lights-on (ZT0–1) that corresponds to a period of peak activity (Fig. 6D). We observed a similar effect in female *Rbf^120a^* homozygotes and *Rbf^120a^/Rbf^14^* trans-heterozygotes, but not in females that were heterozygous for either allele (Fig. 6E), confirming that the above alterations in locomotor activity were caused by mutations in *Rbf*. To characterize these behavioural abnormalities further, we conducted negative geotaxis (climbing) assays.^28^  *Rbf^120a^* hemizygote males displayed significantly lower climbing ability compared with control animals (Supplementary Fig. 4B and C), further indicating that *Rbf* LOF induces significant motor defects in flies.

Given that sleep disturbances were documented in several *RBL2* patients, we tested whether alterations in sleep behaviours were present in *Drosophila Rbf* mutants. *Drosophila* display highly stereotyped sleep patterns, exhibiting high levels of sleep during the middle of the day and night interspersed by peaks of activity centred around lights-on and lights-off.^40^ In 12 h–12 h light–dark conditions, total sleep levels in *Rbf^120a^* hemizygote males did not differ during the day, night or both, compared with controls (Supplementary Fig. 4D–F). However, we observed two clear differences in sleep architecture in *Rbf^120a^* hemizygote males. Firstly, there was a delayed offset of daytime sleep, indicative of loss of increased locomotion prior to lights-off that is normally observed in wild-type flies (Fig. 6F and G and Supplementary Fig. 4G). This motor phenotype, termed ‘evening anticipation’, is driven by the *Drosophila* circadian clock.^41^ Secondly, *Rbf^120a^* males displayed a significant reduction in sleep during the first half of the night (Fig. 6F and H). These phenotypes were observed to a greater degree in *Rbf^120a^* homozygote females (Fig. 6I–K), in which the temporal pattern of sleep was profoundly disrupted, leading to a significant increase in daytime sleep coupled with reduced total nighttime sleep and a loss of evening anticipation (Supplementary Fig. 4H–K). Importantly, we confirmed reduced locomotor activity and altered sleep architecture in *Rbf^120a^* males using a higher-resolution multibeam DAM system (Supplementary Fig. 5), demonstrating that altered motor and sleep behaviours in *Rbf^120a^* males did not reflect different preferences for positions within the DAM system tubes or other confounding behaviours.

The reduced evening anticipation and sleep phenotypes we observed in *Rbf* mutants suggests that Rbf LOF might impair circadian clock function in *Drosophila*. To test this directly, we examined how *Rbf* mutant flies behaved under free-running constant dark (DD) conditions, in which no light cues were present to influence their behaviour. In DD, we found that *Rbf* hypomorphs exhibited a significantly increased circadian period and reduced rhythm strength (Supplementary Fig. 6A–D). Hence, circadian clock defects might contribute to the impaired sleep observed in *Rbf* mutant flies. Overall, these data reveal a conserved role of RBL2/Rbf orthologues in regulating movement and sleep across diverse phyla.

### *Rbf* is highly expressed in adult neurons

**Figure 7:**
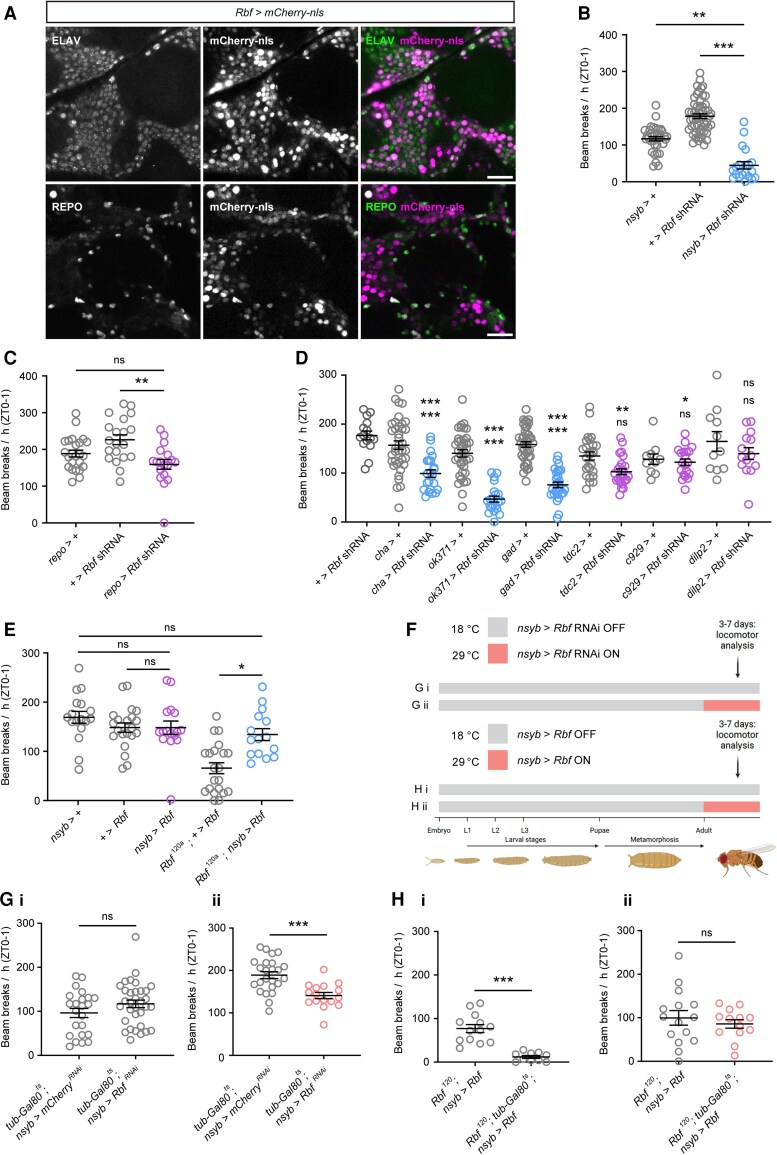
**Adult-stage neuronal expression of Rbf rescues locomotor defects in *Rbf* hypomorphs.** (**A**) *Rbf*-Gal4 driven nuclear mCherry expression in the adult central brain. Neurons and glia are counterstained with antibodies against ELAV and REPO, respectively. Scale bar: 20 µm. (**B**) Pan-neuronal post-mitotic knockdown of *Rbf* severely reduces peak locomotor activity during ZT0–1. *n* = 20–54. (**C**) Knockdown of *Rbf* in glial cells (using *repo*-Gal4 to express *Rbf* shRNA) does not significantly reduce peak locomotor activity during ZT0–1 compared with both driver and transgene alone controls (*n* = 18–24). (**D**) Knockdown of *Rbf* in cholinergic, glutamatergic and GABAergic neurons reduces peak activity during ZT0–1 in adult males. *n* = 11–41. Upper significance notation is relative to *Rbf* shRNA alone controls, lower significance notation is relative to Gal4 driver alone controls. (**E**) Effects of post-mitotic, neuron-specific *Rbf* expression on peak locomotor activity in either wild-type or *Rbf^120a^* hypomorph backgrounds. Data are from adult males. *n* = 15–21. (**F**) Experimental protocol for temperature-induced knockdown and rescue experiments shown in **G** and **H**. (**G**) Quantification of peak activity for: (**i**) control adult male flies kept at non-permissive temperature, *mCherry* (*n* = 33) or *Rbf* (*n* = 23) shRNA expression repressed; and (**ii**) experimental adult male flies maintained at a permissive temperature, *mCherry* (*n* = 24) or *Rbf* (*n* = 16) shRNA expression permitted. [**H**(**i**)] Constitutive suppression of neuronal RBF expression via *tub*-Gal80^ts^ significantly decreases peak locomotor activity in *Rbf^120a^*; *nsyb* > *Rbf* adult males. *Rbf^120a^*, *nsyb* > *Rbf*: *n* = 13; *Rbf^120a^*, *tub*-Gal80^ts^, *nsyb* > *Rbf*: *n* = 10. [**H**(**ii**)] Peak activity in adult male flies that robust RBF expression solely permitted in adult-stage post-mitotic neurons is not significantly different from *Rbf^120a^* hypomorphs with constitutive post-mitotic neuronal expression of RBF. *Rbf^120a^*, *nsyb* > *Rbf*: *n* = 15; *Rbf^120a^*, *tub*-Gal80^ts^, *nsyb* > *Rbf*: *n* = 13. Error bars are the standard error of the mean. *P < 0.05, **P < 0.005, ***P < 0.0005, ns = *P* > 0.05, Kruskal–Wallis test with Dunn’s *post hoc* test (**B** and **E**), one-way ANOVA with Dunnett’s *post hoc* test (**C** and **D**) or unpaired *t*-test with Welch’s correction [**G**(**i**), **G**(**ii**), **H**(**i**) and **H**(**ii**)]. ZT = zeitgeber time.

Given the strong phenotypic similarities between humans and fruit flies harbouring *RBL2/Rbf* LOF mutations, we tested whether we could use *Drosophila* to probe the mechanistic basis of *RBL2*-linked neurodevelopmental defects. Rb proteins are well known for their role in transcriptional repression of cell-cycle-related genes at the G1 to S phase transition.^42^ Hence, it is expected that *Rbf* would be expressed in the developing brain. However, it is unclear whether Rbf also continues to play a role in fully differentiated neurons following cell-cycle exit. To investigate which cells in the nervous system express *Rbf*, we took advantage of a CRISPR-mediated insertion of a Gal4 cassette (CRIMIC insertion) in an *Rbf* intron, which results in expression of Gal4 under control of *Rbf* regulatory sequences (termed *Rbf-*Gal4 hereafter)^43^ (Supplementary Fig. 7A). Crossing these flies with a UAS*-mCherry-*nls line yields expression of nuclear mCherry as a reporter of *Rbf* expression. Examination of *Rbf-*Gal4 activity in larval brains revealed widespread expression, indicating that *Rbf* is indeed broadly expressed in the developing brain (Supplementary Fig. 7B). More surprisingly, adult brains, which do not display appreciable neurogenesis in normal conditions, also exhibited widespread *Rbf*-driven mCherry expression that co-localized with the neuronal marker ELAV (Fig. 7A and Supplementary Fig. 7C). Hence, *Rbf* expression persists in neurons long after terminal cell-cycle exit. In contrast, only a small population of REPO-labelled glial cells expressed mCherry under the control of *Rbf*-Gal4 (Fig. 7A). These findings are consistent with published single-cell RNA-sequencing data showing that *Rbf* is preferentially expressed in post-mitotic neurons relative to glia (Supplemental Fig. 2A).

### *Rbf* knockdown in neurons causes severe behavioural defects

To test directly for an underappreciated role of Rbf in fully differentiated post-mitotic cells, we examined whether reducing Rbf expression in post-mitotic neurons resulted in locomotor defects similar to those observed in constitutive *Rbf* hypomorph flies. We used transgenic RNA interference (RNAi) to deplete *Rbf* specifically in fully differentiated neurons using the *nSyb*-Gal4 driver. Strikingly, deploying the DAM system once more, we found that pan-neuronal knockdown of *Rbf* with a previously verified short hairpin RNA (shRNA)-expressing line^44^ severely reduced peak movement in adult flies (Fig. 7B). To rule out off-target effects, we repeated these experiments using two additional RNA interference lines targeting *Rbf* mRNA. Both constructs similarly reduced peak movement when expressed in post-mitotic neurons (Supplementary Fig. 8A and B). In contrast to neuronal knockdowns, RNA interference-mediated depletion of *Rbf* in glial cells did not significantly reduce peak locomotor activity (Fig. 7C), in accordance with the above observation that *Rbf* expression is less abundant in glia than in neurons (Fig. 7A).

We next used climbing assays to quantify stimulus-induced negative geotaxis. These assays further indicated that flies with reduced Rbf expression in neurons have severe motor defects, showing significantly reduced climbing ability in comparison to controls (Supplementary Fig. 8C). Interestingly, larval locomotion was unchanged in either *Rbf* hypomorphs or following knockdown of *Rbf* in post-mitotic neurons, suggesting that larval neuronal lineages have a differential requirement for Rbf compared with their adult counterparts (Supplementary Fig. 8D and E). Importantly, *Rbf* knockdown in adult post-mitotic neurons did not reduce optic lobe size or induce a measurable increase in neuronal apoptosis (Supplementary Fig. 8F and G). Taken together, these data suggest that *Rbf* plays important neuron-autonomous roles that are essential for adult locomotor behaviour and that are independent of neuronal viability.

### Multiple neuronal subtypes are affected by *Rbf* knockdown

To identify which cell types in the post-mitotic brain are affected by *Rbf* knockdown, we used specific drivers to restrict *Rbf* shRNA expression to genetically defined subsets of neurons. We knocked down *Rbf* in discrete neuronal subtypes, including cholinergic, GABAergic and glutamatergic neurons. Of these, *Rbf* knockdown in glutamatergic neurons (which include *Drosophila* motoneurons) yielded the most significant decline in locomotor activity, as measured using the DAM system (Fig. 7D and Supplementary Fig. 9). *Rbf* knockdown in GABAergic and cholinergic neurons did not significantly decrease overall activity across 24 h (Supplementary Fig. 9). However, in the 1 h period following lights-on (ZT0–1), during which control flies exhibit a peak period of locomotor activity, both cholinergic and GABAergic *Rbf* knockdown flies showed significantly reduced activity (Fig. 7D), indicating a partial perturbation of locomotor capacity. We further tested the motor defects of these flies by conducting climbing assays. These experiments confirmed that reduced *Rbf* expression in glutamatergic, cholinergic or GABAergic neurons resulted in significantly decreased climbing ability compared with controls (Supplementary Fig. 8C). In contrast, *Rbf* knockdown in peptidergic neurons did not perturb overall or peak locomotor activity (Fig. 7D and Supplementary Fig. 9). These data reveal neuronal cell-type-specific effects of Rbf activity on locomotion and suggest that Rbf plays a particularly important role in glutamatergic neurons to promote normal movement in *Drosophila*.

### Post-mitotic restoration of Rbf rescues locomotor defects in *Rbf* hypomorphs

Given that *Rbf* mutants display morphological phenotypes consistent with cell-cycle defects and apoptosis during development, but also behavioural abnormalities that can be induced by knockdown of *Rbf* in post-mitotic neurons, we questioned whether locomotor phenotypes in constitutive *Rbf* hypomorphs were attributable to developmental defects or reduced *Rbf* expression post-neurogenesis (i.e. in post-mitotic neurons). To address this question, we expressed Rbf solely in fully differentiated neurons on the *Rbf* hypomorph background. Interestingly, this manipulation fully rescued the reduced peak activity of *Rbf^120a^* hypomorphs, whereas over-expression of *Rbf* on a wild-type background had no effect on peak locomotor activity (Fig. 7E).

To interrogate more precisely whether Rbf LOF affects adult behaviour owing to developmental perturbations or cell-autonomous activity in adult neurons, we performed complementary adult-stage neuron-specific knockdown and rescue experiments. To do so, we used *tub*-Gal80^ts^, a globally expressed temperature-sensitive inhibitor of Gal4-mediated transgene expression.^45^ In concert with the *nsyb*-Gal4 driver and *Rbf* shRNA or transgenes, this construct allowed us to examine the effects of both adult neuron-specific *Rbf* knockdown on an otherwise wild-type background (Fig. 7F and G) and adult neuron-specific re-expression of Rbf on an *Rbf* hypomorph background (Fig. 7F and H). We initially found that, as expected, peak locomotion in wild-type flies did not significantly differ from controls when *Rbf* shRNA expression in post-mitotic neurons was constitutively repressed at 22°C by active Gal80^ts^ [Fig. 7F and G(i)]. Strikingly, inducing knockdown of *Rbf* in adult-stage neurons by shifting mature experimental flies to 29°C (Gal80 inactive, *Rbf* shRNA expressed) significantly reduced peak locomotion compared with control flies expressing an irrelevant shRNA [Fig. 7F and G(ii)], whereas using the same approach to reduce Rbf expression in post-mitotic neurons only during the pupal stage did not impair locomotion in the resulting adult flies (Supplementary Fig. 10).

In the converse experiment, *Rbf* hypomorphs expressing transgenic Rbf in post-mitotic neurons (*Rbf*^120^, *nsyb > Rbf*) showed significantly higher peak locomotion at 22°C compared with flies of the same genotype but harbouring the repressive *tub*-Gal80^ts^ construct [Fig. 7F and H(i); Gal80 active, transgenic Rbf not expressed]. However, when flies were raised at 22°C, then moved to a permissive temperature of 29°C at the adult stage [Fig. 7F and H(ii); Gal80 inactive, Rbf expressed], we observed no difference in peak activity between these two genotypes, indicating that adult-specific restoration of Rbf expression in post-mitotic neurons was sufficient to rescue locomotor defects in *Rbf* hypomorphs. Taken together, these findings suggest that defects in post-mitotic neuronal function might contribute to morbidities in *RBL2* patients, particularly those associated with motor dysfunction.

## Discussion

RBL2, alongside the RB family members RB1 and RBL1, controls the transition from G1 to S phases of the cell cycle by inhibiting E2F transcription factors, which promote the expression of genes required for DNA synthesis.^46^ Interestingly, of the RB proteins, mutations in *RBL2* are uniquely associated with neurodevelopmental morbidities. However, only six individuals harbouring pathogenic *RBL2* variants have been documented to date,^12-14^ precluding a comprehensive characterization of the genotypic and phenotypic spectrum of this disorder.

Here, we address this knowledge gap by characterizing a cohort of 35 patients from 20 families carrying homozygous or compound heterozygous pLOF variants in *RBL2*. In these patients, we identified 15 novel variants, increasing the number of disease-associated *RBL2* mutations to 20. All variants were predicted as pathogenic or likely pathogenic by *in silico* methods, with variants causing truncations or transcriptional frameshifts likely to cause complete LOF (Supplementary Table 5). Although mutations predicted to perturb the splicing of *RBL2* mRNA might cause LOF via exon skipping, exon extension or intronic retention, further studies are required to determine the degree to which the interaction of RBL2 with chromatin or transcriptional cofactors is disrupted by these variants.

The genetic heterogeneity of RBL2 patients is partly mirrored in their clinical features. Global developmental delay and intellectual disability were observed uniformly across the cohort, and sleep disturbances were noted in all patients for whom data were available. Lack of acquisition of key milestones, such as walking and speech development, and stereotypies were highly prevalent, whereas autism spectrum disorder and aggressive behaviour were observed more variably. Although the present cohort of patients did have facial dysmorphism, our analysis did not suggest a recognizable facial ‘gestalt’.

Common neuroimaging features included cerebral atrophy with an anteroposterior gradient variably associated with white matter volume loss and corpus callosum hypoplasia. In addition, cerebellar atrophy was noted in most *RBL2* patients. We also noted in most cases bilateral faint-to-marked signal changes at the level of the forceps minor, in keeping with an ‘ear-of-the-lynx’ sign. This neuroimaging feature has been reported in hereditary spastic paraplegias (SPG7, SPG11 and SPG15)^47,48^ and other neurodegenerative disorders, including those related to variants in *LNPK*, *CAPN1* and *ATP13A2*.^49-51^ Considering the presence of progressive postnatal microcephaly in most cases, these findings suggest that neurodegeneration is an important feature of this disorder. Indeed, a neurodegenerative component is consistent with our *Drosophila* studies, which suggest that the decreased brain size observed in *Rbf* hypomorphs is driven by an increase in cell death, most probably arising from cell-cycle defects in neuronal precursors and immature neurons. We speculate that the variability in microcephaly among subjects might be attributable to differences in genetic background, which could influence susceptibility to apoptotic mechanisms.

Additionally, three affected individuals were found to have expansile lesions: one orbital mass, one cystic mandibular lesion, and a large mass extending from the III ventricular floor to the prepontine cisterns. This is consistent with previous studies pointing to the potential role of RBL2 dysfunction in the evolution of cancer^52^ and supports the premise that RBL2 plays dual roles in tumour suppression and neuronal differentiation and survival, thus providing further connection between tumorigenic processes and neurodevelopmental disorders.^53^ Overall, both the clinical and neuroradiological findings underscore substantial intrafamilial and interfamilial variations in phenotypic expressions and severity, revealing considerable complexity within and between families.

Similarly to RBL2 patients, we find that LOF in the *Drosophila* RBL2 homologue Rbf leads to reduced brain growth alongside developmental delay, perturbed movement and disrupted sleep. Such phenotypic concordances point to deeply conserved neural roles for RBL2 homologues across phyla. Indeed, the sleep defects observed in RBL2 patients and the altered sleep onset and circadian rhythms in *Drosophila Rbf* mutants suggest previously unrecognized roles for RBL2/Rbf in regulating sleep timing. Furthermore, we uncovered an unexpected movement-promoting role for *Drosophila* Rbf in adult post-mitotic neurons, advocating a model in which Rbf (and, by extension, RBL2) acts sequentially in neural precursors and post-mitotic neurons to promote normal brain morphology and locomotor activity, respectively (Supplementary Fig. 11).

How Rbf influences gene expression in post-mitotic neurons is unclear. Rbf has been shown to modulate gene expression outside of its canonical function in repressing cell-cycle genes; for example, in controlling muscle differentiation.^54^ Thus, it is conceivable that Rbf coordinates undefined gene expression programmes in mature neurons. Alternatively, via its canonical role, Rbf might sustain the epigenetic environment that maintains cell-cycle gene repression in neurons.^46^ Indeed, a recent study showed that chromatin might remain accessible at cell-cycle genes in post-mitotic neurons, with expression of E2F activator complexes sufficient to re-activate cell-cycle gene expression.^55^ Cell-cycle genes have been shown previously to act in neurons to regulate sleep and circadian rhythms in *Drosophila*.^56,57^ Hence, de-repression of cell-cycle genes following RBL2/Rbf LOF could plausibly perturb neuronal development, intrinsic excitability or synaptic release, leading to defects in movement, sleep and other neurological features. These hypotheses can now be tested using the genetic tools available in *Drosophila*.

Our work has limitations that can be addressed through future studies. As noted above, it remains unclear whether all *RBL2* variants in our patient cohort cause complete LOF. Generating corresponding knock-in *Drosophila* or vertebrate models could help to address this question and enhance the understanding of genotype–phenotype correlations in RBL2 patients. Furthermore, although our study indicates conserved roles of human RBL2 and *Drosophila* Rbf, it is possible that functional divergence has occurred between these species. The human genome contains three Rb genes and eight genes encoding interacting E2F transcription factors,^58^ in comparison to two Rb and E2F loci in *Drosophila*. Therefore, the greater complexity of the human RB/E2F network could result in altered biological outcomes. Indeed, both *Drosophila* and mice harbouring null alleles of *Rbf/RBL2* are embryonic lethal,^15,36^ in contrast to human patients homozygous for truncating *RBL2* variants. Identifying the neuronal circuits in which Rbf acts to promote movement, circadian rhythms and sleep in *Drosophila* might also suggest key neuronal cell types disrupted by RBL2 LOF in human patients. Additionally, although our study aims to expand and delineate the full phenotypic spectrum of *RBL2-*related disorder, further studies will be needed to characterize fully some aspects of the disorder, such as sleep disturbances, autistic features and other behavioural abnormalities. Finally, although seizures in some RBL2 patients were ameliorated by anti-epileptic drugs (Supplementary Table 2), treatments for the majority of RBL2 patient symptoms are lacking. However, our *Drosophila* studies raise the possibility that some patient phenotypes, particularly relating to movement defects, might be treatable acutely through gene therapy approaches to restore RBL2 expression in neurons. The generation of vertebrate models of *RBL2* disorder harbouring partial LOF alleles will be an important step towards testing this clinically relevant hypothesis.

## Supplementary Material

awae363_Supplementary_Data

## Data Availability

The authors declare that the data supporting the findings of this study are available within the paper and its Supplementary material.
