# Supplementary material for: Clinical and genetic characterization of a progressive RBL2-associated neurodevelopmental disorder
Source: Brain. 2024 Dec 18;148(4):1194–211. doi: 10.1093/brain/awae363 (PMC11967543; doi:10.1093/brain/awae363)
Supplement: awae363_Supplementary_Data [file awae363_supplementary_data.zip › brain-2024-01175-File010.pdf]

Clinical and genetic characterisation of a progressive RBL2-associated neurodevelopmental disorder

Gabriel N. Aughey[1*], Elisa Cali[2*] , Reza Maroofian[2*], Maha S. Zaki[3], Alistair T Pagnamenta[4], Zafar Ali[5], Uzma Abdulllah[6], Fatima Rahman[7], Lara Menzies[8], Anum Shafique[9], Mohnish Suri[10,11], Emmanuel Roze[12], Mohammed Aguennouz[13], Zouiri Ghizlane[14], Saadia Maryam Saadi[15], Ambrin Fatima[16] ,Huma Arshad Cheema[17], Muhammad Nadeem Anjum[17], Godelieve Morel[18], Stephanie Robin[18], Robert McFarland[19,20], Umut Altunoglu[21], Verena Kraus[22], Moneef Shoukier[23], David Murphy[24], Kristina Flemming[25], Hilde Yttervik[26], Hajar Rhouda[13], Gaetan Lesca[27], Nicolas Chatron[27], Massimiliano Rossi[27], Bibi Nazia Murtaza[28], Mujaddad Ur Rehman[28], Jenny Lord[29], Edoardo Giacopuzzi[30], Azam Hayat[31], Muhammad Siraj[32], SYNAPS Study Group[2], Genomics England Consortium[33], Reza Shervin Badv[34], Go Hun Seo[35], Christian Beetz[36], Hülya Kayserili[21], Yamna Krioulie[13], Wendy K. Chung[37], Sadaf Naz[9], Shazia Maqbool[7], Kate Chandler[38], Christopher Kershaw[38], Thomas Wright[38,39] Siddharth Banka[38,39], Joseph G. Gleeson[40,41], Jenny C. Taylor[4], , Stephanie Efthymiou[2], Shahid Mahmood Baig[16,42], Mariasavina Severino[43], James E.C. Jepson[1#], Henry Houlden[2#]

Supplementary Figures 1-11

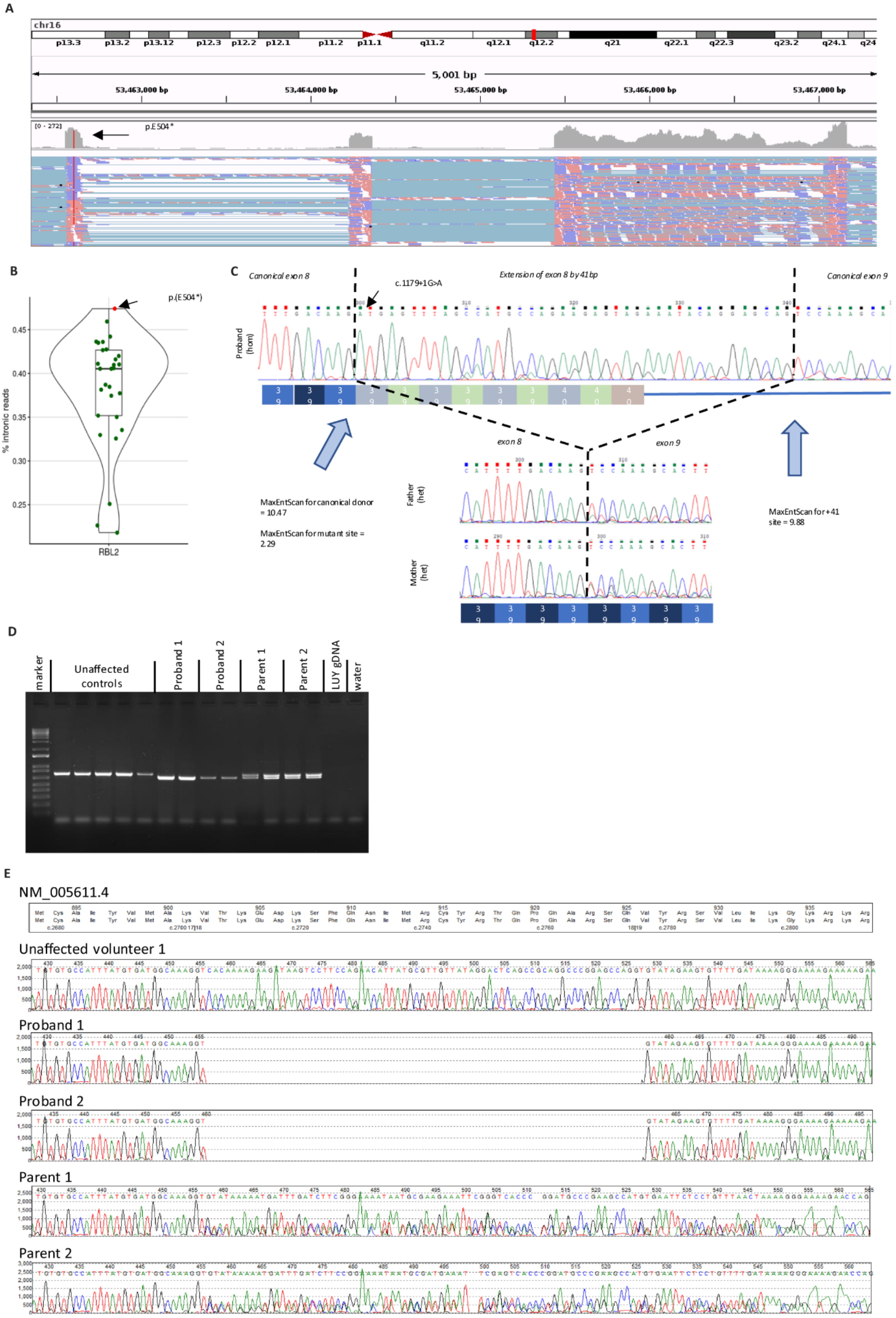
A
chr16
p13.3
p13.2
p13.12
p12.3
p12.2
p12.1
p11.2
p11.1
q11.2
q12.1
q12.2
q21
q22.1
q22.3
q23.2
q24.1
q24
5,001 bp
53,463,000 bp
53,464,000 bp
53,465,000 bp
53,466,000 bp
53,467,000 bp
[0 - 272]
p.E504*
B
p.(E504*)
% intronic reads
0.45
0.40
0.35
0.30
0.25
RBL2
C
Canonical exon 8
c.1179+1G>A
Extension of exon 8 by 41bp
Canonical exon 9
Proband (hom)
exon 8
exon 9
Father (het)
Mother (het)
MaxEntScan for canonical donor = 10.47
MaxEntScan for mutant site = 2.29
MaxEntScan for +41 site = 9.88
D
marker
Unaffected controls
Proband 1
Proband 2
Parent 1
Parent 2
LUY gDNA
water
E
NM_005611.4
Unaffected volunteer 1
Proband 1
Proband 2
Parent 1
Parent 2

Supplementary Fig. 1. A. IGV screenshot showing RNAseq data for the proband in Family F1. The c.1510G>T variant in exon 11 was observed in 179/179 reads. Elevated intron retention is seen particularly for intron 13. **B.** Percentage of intronic reads in *RBL2*. **C.** Sanger sequencing of RT-PCR products indicates that the c.1179+1G>A variant (Family F7) results in extension of exon 8 by 41 bp. **D.** Agarose gel of PCR of exons 15 to 20 of RBL2 NM_005611.4 from cDNA derived from five unaffected volunteers, the probands and parents of Family F16. **E.** Sanger sequencing trace of the probands and parents confirming exon 18 exclusion from RBL2 in the probands and the expected heterozygous balance in the parents. The Sanger trace from volunteer 1 is included as a control.

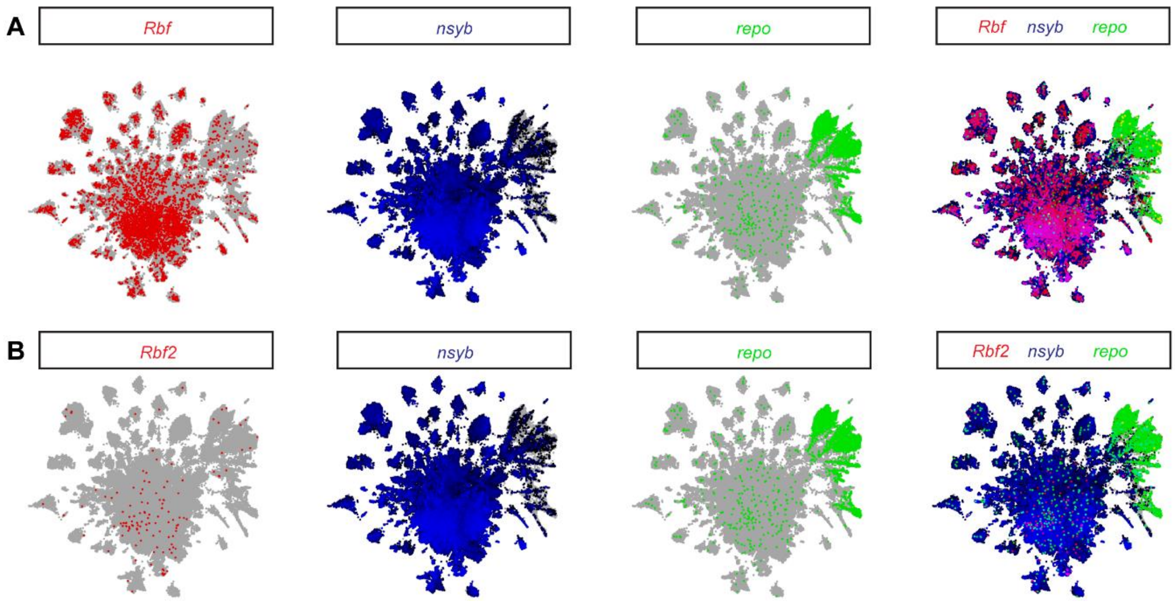

Supplementary Fig. 2. A-B. Single-cell RNAseq-derived t-distributed stochastic neighbor embedding (t-SNE) plots showing *Rbf* (A) and *Rbf2* (B) expression, alongside markers for glial cells (*repo*) and post-mitotic neurons (*nsyb*), in the adult fly brain. Gene expression across adult brain cells is illustrated using *SCope*.

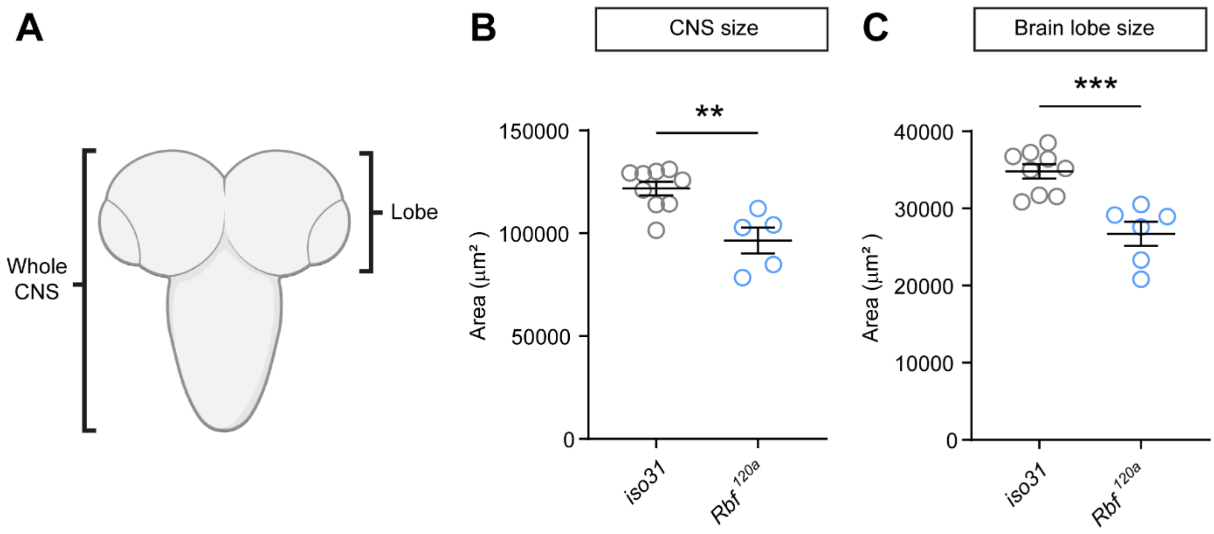

Supplementary Fig. 3. A. Schematic illustrating larval CNS anatomy. **B.** Quantification of total brain or **C.** optic lobe size of 3rd instar larval CNS in iso31 (n = 9) and *Rbf*^{120a} hemizygotes (n = 6). Error bars: SEM. ** p<0.005, *** p< 0.0005.

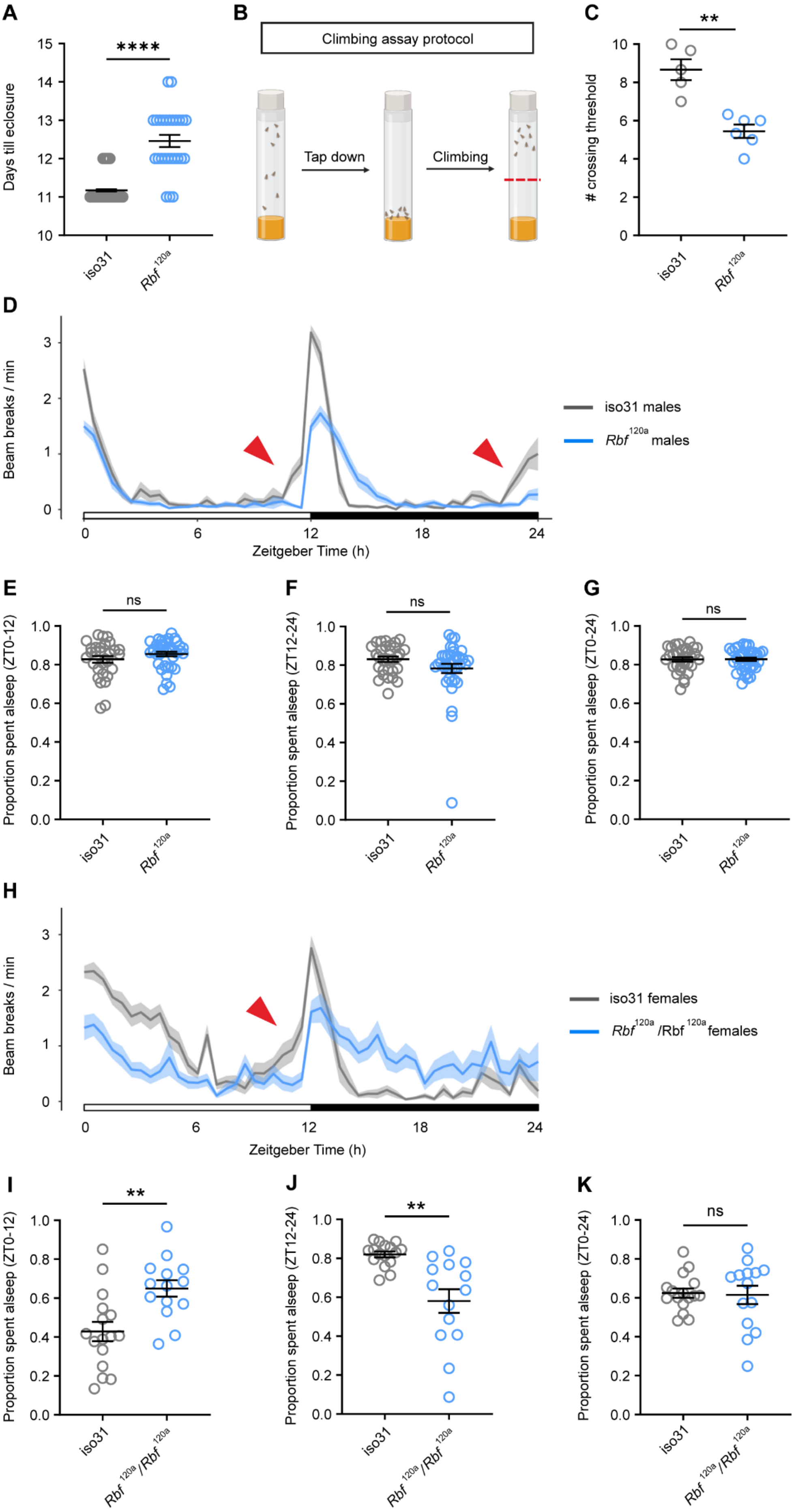
A
Days till eclosure
****
iso31
Rbf 120a
B
Climbing assay protocol
Tap down
Climbing
C
crossing threshold
**
iso31
Rbf 120a
D
Beam breaks / min
Zeitgeber Time (h)
iso31 males
Rbf 120a males
E
Proportion spent alseep (ZT0-12)
ns
F
Proportion spent alseep (ZT12-24)
ns
G
Proportion spent alseep (ZT0-24)
ns
H
Beam breaks / min
Zeitgeber Time (h)
iso31 females
Rbf 120a /Rbf 120a females
I
Proportion spent alseep (ZT0-12)
**
J
Proportion spent alseep (ZT12-24)
**
K
Proportion spent alseep (ZT0-24)
ns
iso31
Rbf 120a /Rbf 120a

Supplementary Fig. 4. A. Days to eclosion per fly for iso31 control (n = 201) and *Rbf*^{120a} hemizygotes (n = 26). **B.** Schematic showing protocol to assess climbing ability in adult flies. **C.** Number of flies (out of n = 10) passing a given threshold (see Materials and Methods) as a measure of climbing ability. Control adult males: n = 5 replicates, *Rbf*^{120a} hemizygotes: n = 6 replicates**. D.** Locomotor activity across the day/night cycle in control and *Rbf*^{120a} males. Red arrows point to clear loss of both evening and morning anticipation in *Rbf*^{120a} males, defined by the absence of an increase in locomotor activity prior to lights-off and lights-on. n = 31 iso31 males and 38 *Rbf*^{120a} males. **E-G.** Proportion of time spent asleep across the day (C), night (D), or entire 24 h period (E), in control and *Rbf*^{120a} males. **F. H.** Locomotor activity across the day/night cycle in control and *Rbf*^{120a} females. Red arrows point to loss evening anticipation in *Rbf*^{120a} females. n = 16 iso31 and 14 *Rbf*^{120a} females. **I-K**: Proportion of time spent asleep across the day (G), night (H), or entire 24 h period (I), in control and *Rbf*^{120a} females. **J.** Error bars: SEM. ns – p> 0.05, ** p<0.005, unpaired t-test with Welch's correction (B, E, G, H, I) or Mann-Whitney U-test (C, D).

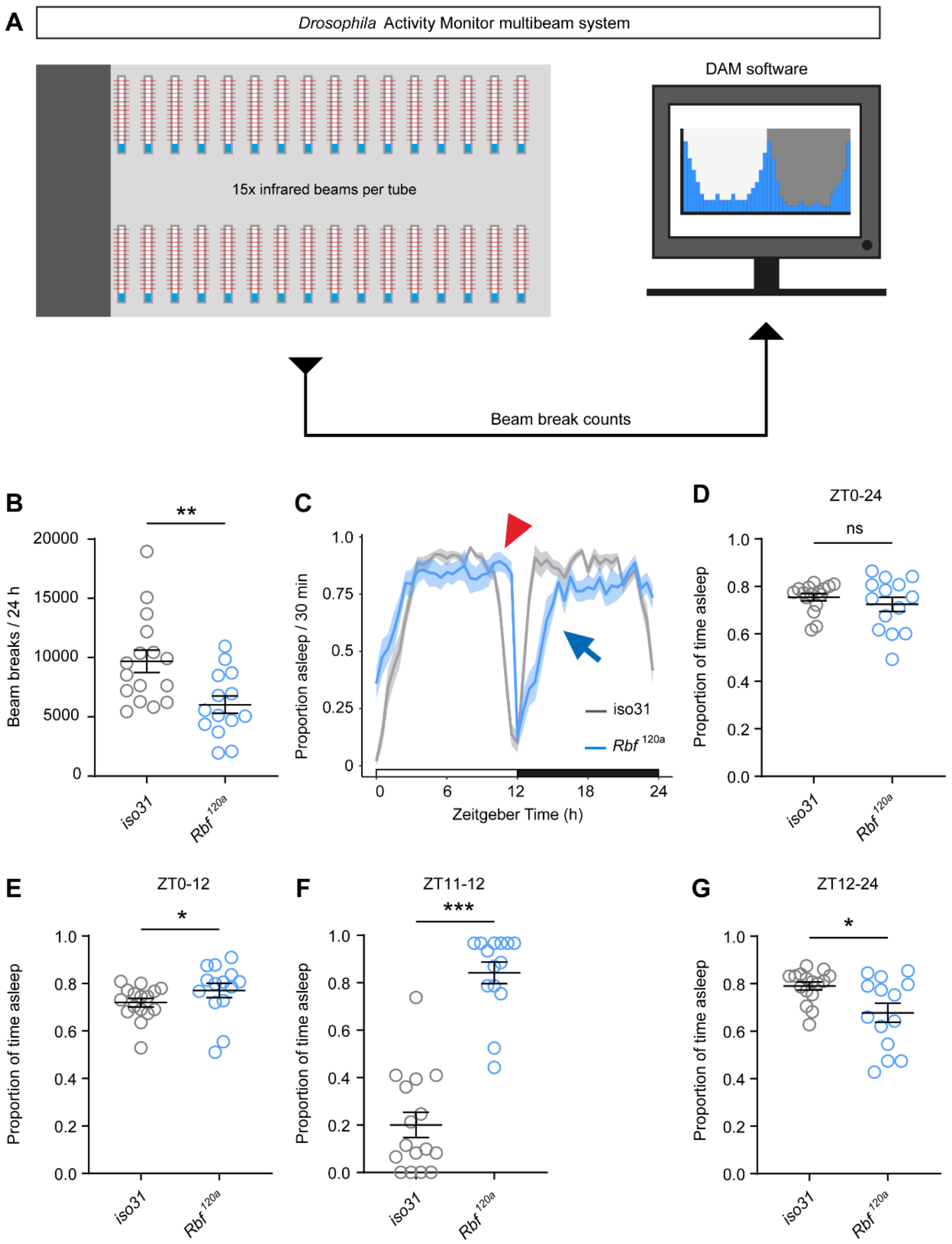

Supplementary Fig. 5. A. Schematic illustrating multibeam DAM system. **B.** DAM activity in *Rbf*^{120a} hemizygotes (n = 16) and controls (iso31; n = 14) across a 24 h period. **C.** Sleep traces of control (iso31) and *Rbf*^{120a} hemizygote males showing the proportion of time spent asleep during 30 min windows across a 12 h light: 12 h dark period. Note the clear loss of evening anticipation prior to lights off (red arrow), and the delayed onset of night sleep (blue

arrow). **D-G**. Quantification of the proportion of time spend sleeping in control (iso31, n = 14) and *Rbf*^{120a} hemizygote (n = 16) males across 24 h (ZT0-24, D), during the day (ZT0-12, E), the final hour of the day (ZT11-12, F), and the night (ZT12-24, G). Error bars: SEM. * p<0.05, ** p<0.005, *** p<0.0005, unpaired t-test with Welch’s correction (C) or Mann-Whitney U-test (D-G).

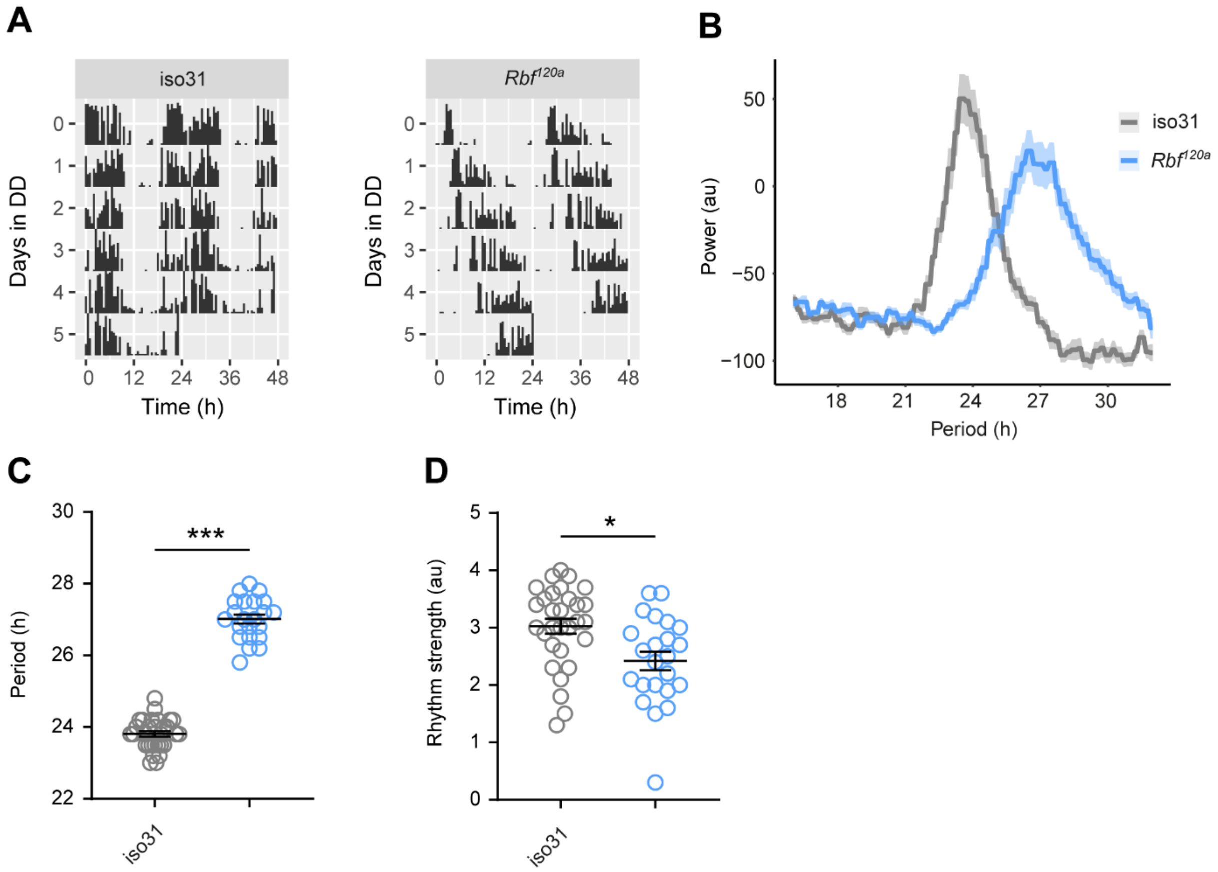

Supplementary Fig. 6. A. Representative double-plotted actograms illustrating circadian behaviour in iso31 controls and *Rbf*^{120a} hemizygotes. **B.** Periodogram showing mean circadian period length and rhythm power. **C.** Dot plot representing period length per-fly in iso31 control (n = 16) and *Rbf*^{120a} hypomorph (n = 16) adult male flies. **D.** Dot plot representing rhythm power per fly. Error bars: SEM. * p<0.05, *** p<0.0005, unpaired t-test with Welch's correction.

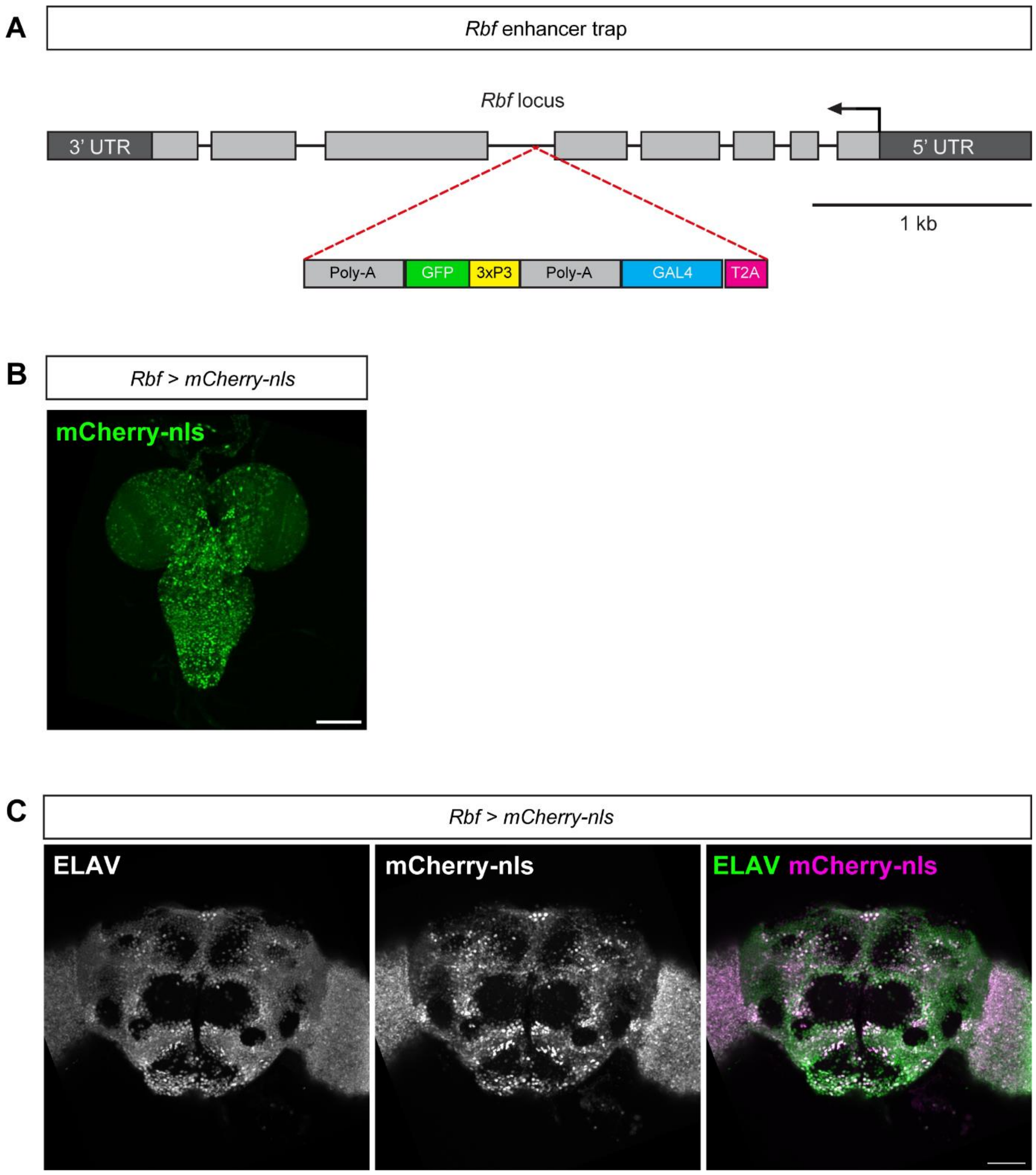

Supplementary Fig. 7. A. Schematic representation of the CRIMIC insertion in the *Rbf* locus, which allows for *Rbf*-dependent Gal4 reporter expression. **B-C.** *Rbf*-Gal4 driven nuclear mCherry expression in the *Drosophila* larval (B) and adult (C) brain. Adult neuronal nuclei are counterstained with an antibody against ELAV in (C). Scale bar = 100 µm.

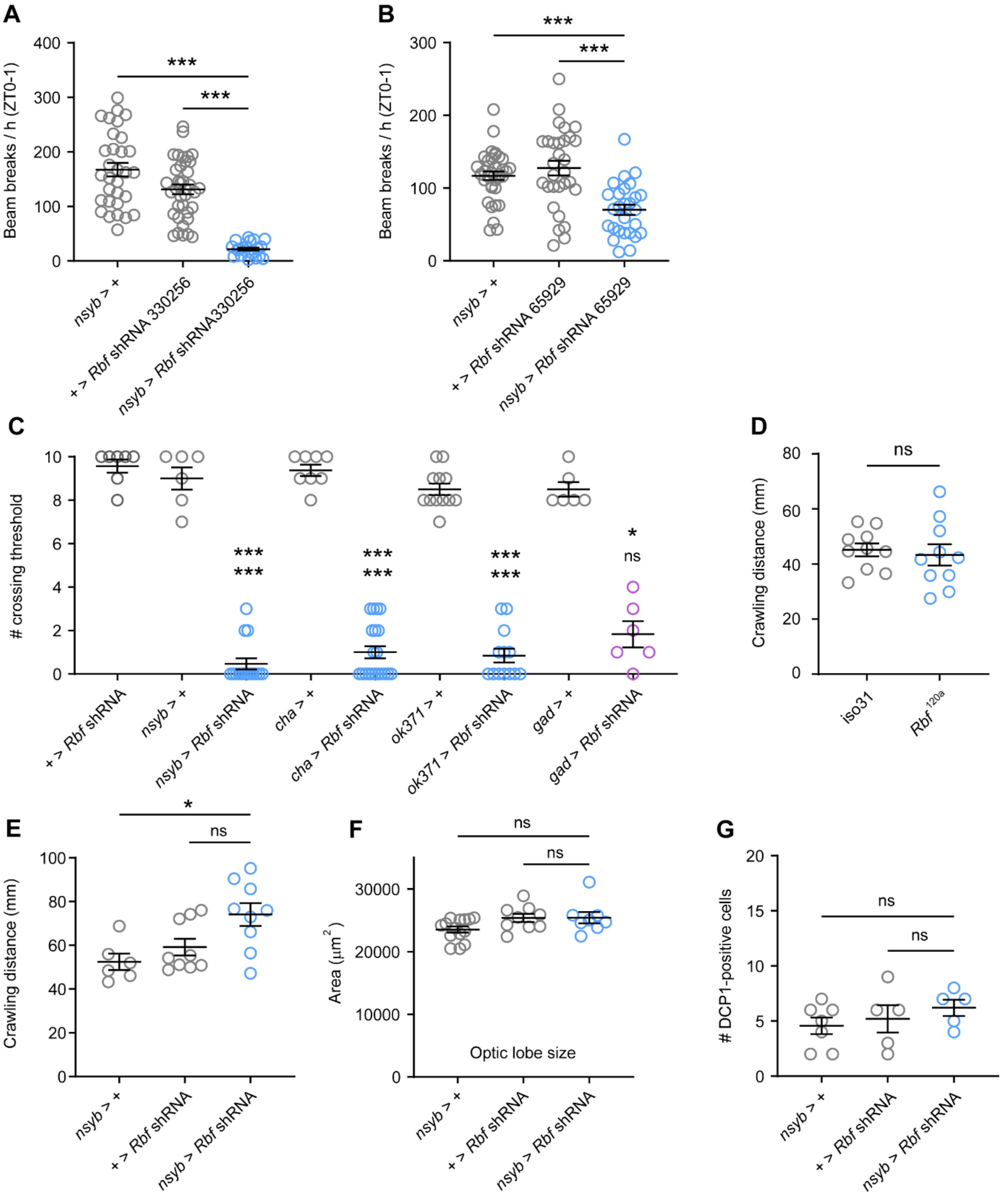

Supplementary Fig. 8. A-B. Expression in post-mitotic neurons of two distinct shRNAs (denoted as 330256 (A) and 65929 (B)) targeting *Rbf* mRNA reduces peak activity in adult Drosophila males. A: n = 20-37. B: n = 27-35. **C.** Number of flies (out of n = 10) passing a given threshold as a measure of climbing ability. n = 6-20 replicates. **D-E.** Crawling ability in control or *Rbf*^{120a} hypomorph 3rd instar larvae (D) or larvae subject to *Rbf* knockdown in post-

mitotic neurons versus driver/transgene alone controls (E). D: n = 10 per genotype. E: n = 6-9. **F-G.** Mean optic lobe size (F) and number of apoptotic (DCP1-positive) cells in adult male brains subject to *Rbf* knockdown in post-mitotic neurons versus driver/transgene alone controls. F: n = 8-14. G: n = 5-7. Error bars: SEM. * $p < 0.05$, ** $p < 0.005$, *** $p < 0.0005$, ns – $p > 0.05$, one-way ANOVA with Dunnett post-doc test (A, B, G), Kruskal-Wallis test with Dunn’s post-hoc test (C, E, F), or unpaired t-test with Welch’s correction (D).

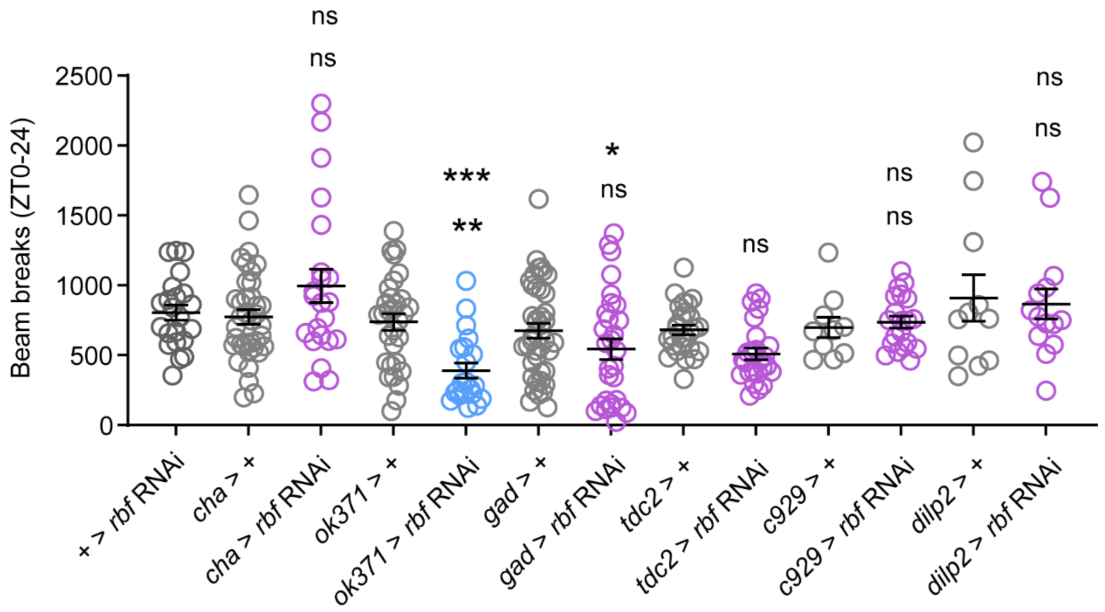

Supplementary Fig. 9. Knockdown of *Rbf* in glutamatergic neurons, reduces total activity during ZT0-24 in adult males. n = 11-41. Upper significance notation is relative to *Rbf* shRNA alone controls, lower significance notation is relative to Gal4 driver alone controls. Error bars: SEM. * $p < 0.05$, ** $p < 0.005$, *** $p < 0.0005$, ns – $p > 0.05$, Kruskal-Wallis test with Dunn's post-hoc test.

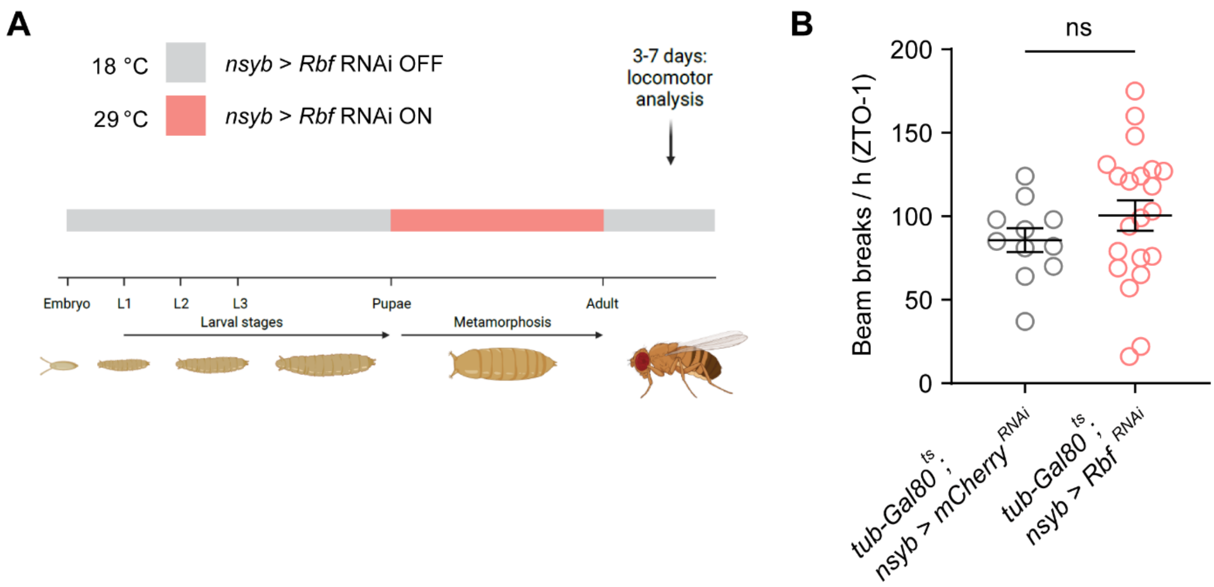

Supplementary Fig. 10. A. Schematic illustrating experimental paradigm in which *Rbf* expression is inhibited by Gal80 specifically during pupal stages. **B.** Peak locomotor activity is not significantly affected (ZT0-1) in flies with *Rbf* knocked down in neurons during pupal development. Error bars: SEM. ns – p> 0.05.

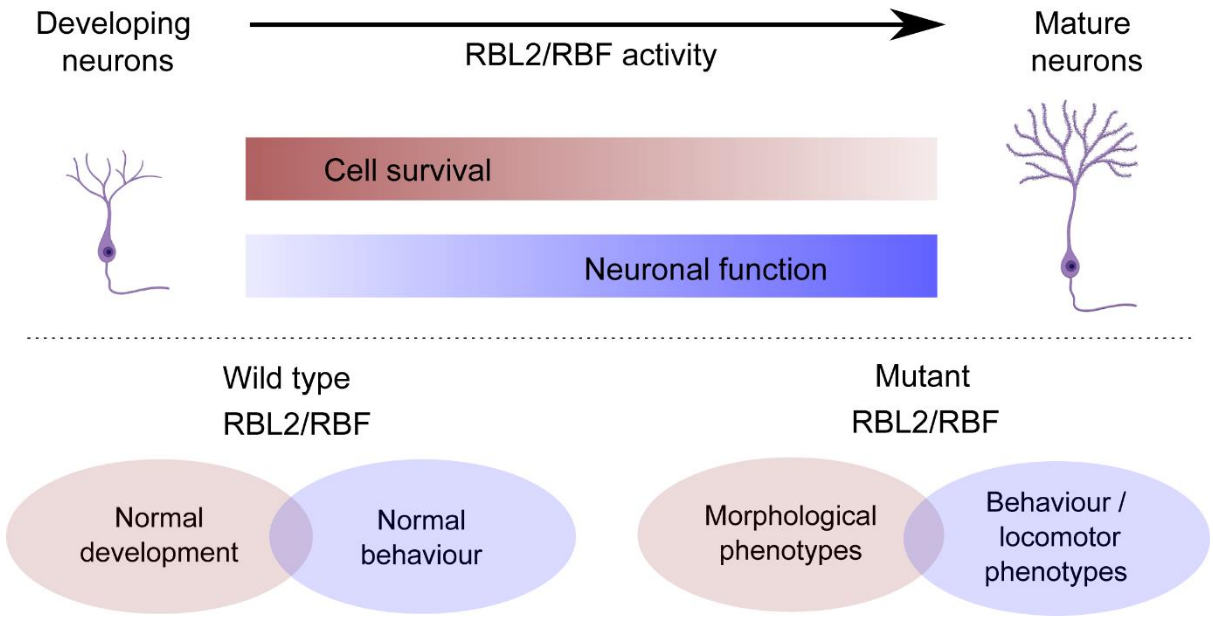

Supplementary Fig. 11. Model of dynamic changes in RBF function in developing and adult neurons.

Video legend

Video illustrating the stereotypies observed in patients with a neurodevelopmental encephalopathy due to biallelic *RBL2* pathogenic variants. Segment 1 (patient from family F3) shows orofacial stereotypies with teeth grinding associated with hand clasping stereotypies. Segment 2 (from family F11) shows multiple hands stereotypies comprising hand squeezing, hand mouthing, finger wiggling and finger tapping, and multiple orofacial stereotypies with a prominent involvement of the tongue.
