# Supplementary material for: Clinical and genetic characterization of a progressive RBL2-associated neurodevelopmental disorder
Source: Brain. 2024 Dec 18;148(4):1194–211. doi: 10.1093/brain/awae363 (PMC11967543; doi:10.1093/brain/awae363)
Supplement: awae363_Supplementary_Data [file awae363_supplementary_data.zip › brain-2024-01175-File011.pdf]

Genotype	Description	Source
Rbf^{120a}/FM7i, P{ActGFP}JMR3	*Rbf* hypomorphic allele.	BDSC # 81612
Rbf^{14} w^{1118}/FM7c	*Rbf* null allele	BDSC # 7435
y^1 sc^* v^1 sev^{21}; P{TRiP.HMS03004}attP2/TM3, Sb1	RNAi targeting *Rbf.*	BDSC # 36744
P{VSH330256}attP40	RNAi targeting *Rbf.*	VDRC # v330256
y^1 sc^* v^1 sev^{21}; P{TRiP.HMC06195}attP2	RNAi targeting *Rbf.*	BDSC # 65929
$w^{1118}{}_{iso}$; 2_{iso}; 3_{iso}	Isogenised control strain (iso31)	Gift from Prof. Kyunghee Koh
y^1 TI{CRIMIC.TG4.2}$^{RbfCR00505-TG4.2}$ w^*/FM7h	Expresses Gal4 under the control of *Rbf* regulatory sequences.	BDSC # 78934
w^{1118}; P{GawB}$VGlut^{OK371}$	Expresses Gal4 in glutamatergic neurons.	Gift from Prof. Kyunghee Koh
cha-Gal4	Expresses Gal4 in cholinergic neurons.	Gift from Prof. Kyunghee Koh
Gad-Gal4	Expresses Gal4 in GABAergic neurons.	Gift from Prof. Kyunghee Koh
Dilp2-Gal4	Expresses GAL4 in insulinergic cells.	Gift from Prof. Kyunghee Koh
C929-Gal4	Expresses GAL4 in peptidergic cells.	Gift from Prof. Kyunghee Koh
Tdc2-Gal4	Expresses GAL4 in aminergic cells.	BDSC # 9313
w^*; UAS-Rbf	Expressed *Rbf* under the control of UAS sequence.	BDSC # 50746
Rbf^{120a}/FM7i ; UAS-Rbf / CyO	Allows for targeted expression of Rbf in *Rbf* hypomorph background.	This study
tub-$Gal80^{ts}$	Ubiquitous temperature sensitive expression of the Gal80 repressor	Gift from Prof. Kyunghee Koh
nSyb-Gal4	Expresses Gal4 pan-neuronally.	BDSC#51635
tub$Gal80^{ts}$; nSyb-Gal4	Allows for pan-neuronal temperature inducible expression.	This study
UAS-mCherry-NLS	Fluorescent reporter.	BDSC# 38424
y[1] sc[*] v[1] sev[21]; P{y[+t7.7] v[+t1.8]=UAS-mCherry.VALIUM10}attP2	RNAi control.	BDSC# 35787
